# Disruption of ARID1B Recruitment to the Nuclear Pore Complex as a New Anticancer Therapeutic Strategy

**DOI:** 10.1002/advs.202415585

**Published:** 2025-07-16

**Authors:** Olena Odnokoz, Anupam Banerjee, Xin Cui, Lidan Zeng, Amad Uddin, Christopher Li, Yueming Zhu, Mengyuan Zhang, Xiaodong Lu, Nagendra S. Yarla, Lu Wang, Jindan Yu, Jonathan C. Zhao, Ivet Bahar, Yong Wan

**Affiliations:** ^1^ Department of Pharmacology and Chemical Biology Emory University School of Medicine Atlanta GA 30322 USA; ^2^ Winship Cancer Institute Emory University School of Medicine Atlanta GA 30322 USA; ^3^ Laufer Center for Physical and Quantitative Biology Stony Brook University Stony Brook NY 11794 USA; ^4^ Department of Biochemistry and Molecular Biology and Institute of Bioinformatics University of Georgia Athens GA 30602 USA; ^5^ Department of Urology Emory University School of Medicine Atlanta GA 30322 USA; ^6^ Department of Biochemistry and Molecular Genetics Northwestern University Feinberg School of Medicine Chicago IL 60611 USA; ^7^ Department of Human Genetics Emory University School of Medicine Atlanta GA 30322 USA; ^8^ Department of Biochemistry and Cell Biology Renaissance School of Medicine Stony Brook University Stony Brook NY 11794 USA

**Keywords:** ARID1B, carcinogenesis, KPNA2, KPNB1, nuclear translocation, RANBP2

## Abstract

Triple‐negative breast cancer (TNBC), a highly aggressive subtype, currently lacks potent targeted therapies. ARID1B, a key SWI/SNF chromatin remodeling complex subunit, is linked to high‐grade malignancies and poor prognosis, making it a potential biomarker and therapeutic target. However, its function and regulation remain unclear. Here, it is found that uncontrolled accumulation of ARID1B and its dysregulated nuclear import promoted oncogenesis and drug resistance. ARID1B negatively regulates ARID1A, impairing SWI/SNF‐mediated tumor suppression and enhancing tumor survival. Using protein complex purification and mass spectrometry, the KPNA2–KPNB1–RANBP2 protein cascade is identified as critical for facilitating ARID1B nuclear import. Replacing R1518, H1519, and D1522 residues on ARID1B with T1518, G1519, and G1522 attenuates the ARID1B–KPNA2/KPNB1 interaction, preventing recruitment of ARID1B to the nuclear pore complex (NPC). Pharmacologically inhibiting KPNB1 suppressed ARID1B translocation, limiting its nuclear levels. In TNBC mouse models, ARID1B knockout (KO) significantly reduces tumor growth and enhances PARP inhibitor efficacy. Collectively, these findings uncover an undocumented mechanism for ARID1B nuclear translocation and reveal that blockade of ARID1B nuclear translocation can be a new therapeutic strategy for TNBC.

## Graphical abstract

This study identifies ARID1B as a chromatin‐bound driver of tumor growth in TNBC. ARID1B impairs ARID1A function and directly activates oncogenic programs through SWI/SNF remodeling. Its nuclear localization, mediated by the KPNA2–KPNB1–RANBP2 import machinery, is essential for its tumor‐promoting activity. Disrupting ARID1B nuclear import suppresses tumor progression and enhances sensitivity to PARP inhibitors.

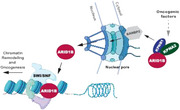

## 1 Introduction

Despite recent advances in diagnosis and treatment, breast cancer continues to lead among malignant neoplasms in women and remains the second leading cause of death.^[^ ^1^ ^]^ TNBC is a subtype that lacks expression of the estrogen receptor (ER), progesterone receptor (PR), and human epidermal growth factor receptor 2 (HER2), accounting for 15% – 20% of all breast cancers.^[^ ^2^, ^3^ ^]^ TNBC is the most invasive, highly metastatic, and chemotherapy‐resistant subtype among breast cancers.^[^ ^4^, ^5^ ^]^ Treatment options for TNBC are limited and struggle to address recurrent and unresectable tumors.^[^ ^5^, ^6^ ^]^ Currently, both early‐stage and advanced TNBC are primarily treated with highly cytotoxic chemotherapies.^[^ ^7^, ^8^ ^]^ Although therapeutic advancements, such as immune‐targeted approaches, PARP inhibitors, and antibody‐drug conjugates (ADCs), have expanded beyond traditional chemotherapy, effective targeted therapies for TNBC are still lacking.^[^ ^9^ ^]^ Immunotherapy offers a promising alternative due to TNBC's high immunogenicity. Pembrolizumab, for example, has been approved for early‐stage TNBC, demonstrating the potential of immune‐based approaches.^[^ ^8^ ^]^ However, resistance remains a significant challenge, with 60%–85% of TNBC patients showing resistance to immunotherapy.^[^ ^10^ ^]^ Thus, it is crucial to decipher the in‐depth molecular events underlying TNBC malignancy and identify new therapeutic targets for drug design. Our search for such targets, using big data analyses and pathological validation based on patient specimens, has drawn our attention to ARID1B as an important factor in mediating tumor growth and pathogenesis.

ARID1B belongs to the AT‐rich Interaction Domain (ARID) subfamily of proteins and shares high sequence identity with its paralogue ARID1A.^[^ ^11^, ^12^, ^13^ ^]^ It is an essential component of the SWI/SNF chromatin remodeling complexes, also known as the BAF complexes, plays a crucial role in assembling the fully functional complex, and is vital for controlling cell proliferation, differentiation, apoptosis, and genome stability. ARID1B achieves this by linking the BAF complex to chromatin and facilitating the recruitment of regulatory factors to DNA.^[^ ^12^, ^13^, ^14^, ^15^, ^16^, ^17^, ^18^, ^19^ ^]^ Both mutations and dysregulation of genes encoding components of the SWI/SNF complexes, including ARID1B, have been implicated in various malignancies, including breast cancer.^[^ ^13^, ^20^, ^21^, ^22^, ^23^, ^24^ ^]^ Results from recent pathological studies based on patient cohorts indicated that elevated accumulation of ARID1B in breast tumors is associated with poor prognosis.^[^ ^25^, ^26^ ^]^

The interplay between ARID1A and ARID1B in cancer has been a subject of research interest, showing functional redundancy in some contexts. However, they also have distinct roles and alterations in either gene can lead to different consequences in cancer. In early stages or under homeostatic conditions, ARID1A and ARID1B, when functioning properly within the BAF complex, contribute to the suppression of tumor progression by regulating gene expression through chromatin remodeling. However, in advanced tumors, loss of ARID1A due to mutations or its downregulation can lead to a shift in SWI/SNF complex function from tumor‐suppressive to oncogenic. This is where ARID1B may become dominant over mutated ARID1A, promoting tumor survival.^[^ ^13^, ^22^, ^23^, ^24^, ^27^ ^]^ The mutual exclusion observed between ARID1A and ARID1B in tumor cells highlights their complex interplay and the importance of proper regulation in maintaining cellular homeostasis versus promoting tumorigenesis.^[^ ^11^, ^13^, ^18^, ^23^, ^24^, ^25^ ^]^ Ongoing research aims to further elucidate the precise mechanisms regulating ARID1A and ARID1B, including transcriptional control, nuclear translocation, and their roles within the SWI/SNF complex in chromatin remodeling and other functions. This understanding is crucial for the development of targeted therapies and biomarkers for cancers associated with the dysregulation of these chromatin remodelers.

In this study, using a multidisciplinary approach that included clinical cohorts of human breast cancer patients, we dissected the role of ARID1B in breast tumor progression and drug resistance. We identified KPNA2–KPNB1–RANBP2 as the nuclear import cascade governing ARID1B translocation from the cytoplasm to the nucleus, utilizing mass spectrometry and molecular simulations. Additionally, we recapitulated the dynamic processes of ARID1B recognition by KPNA2, recruitment by KPNB1, and its delivery to the NPC. Replacement of R1518, H1519, and D1522 with T1518, G1519, and G1522 hindered the interaction between KPNA2 and ARID1B. Pharmacological inhibition of KPNB1 blocked ARID1B translocation from the cytosol to the nucleus, impeding tumor growth. Finally, we discovered that by suppressing ARID1B function via blocking its nuclear import or via KO of the ARID1B gene in vivo, we not only inhibited tumor growth but also augmented the therapeutic efficacy of PARP inhibitors in treating TNBC tumors.

## 2 Results

### 2.1 Elevated Expression of ARID1B Correlates with a Poor Prognosis of Breast Cancer

**Figure 1:**
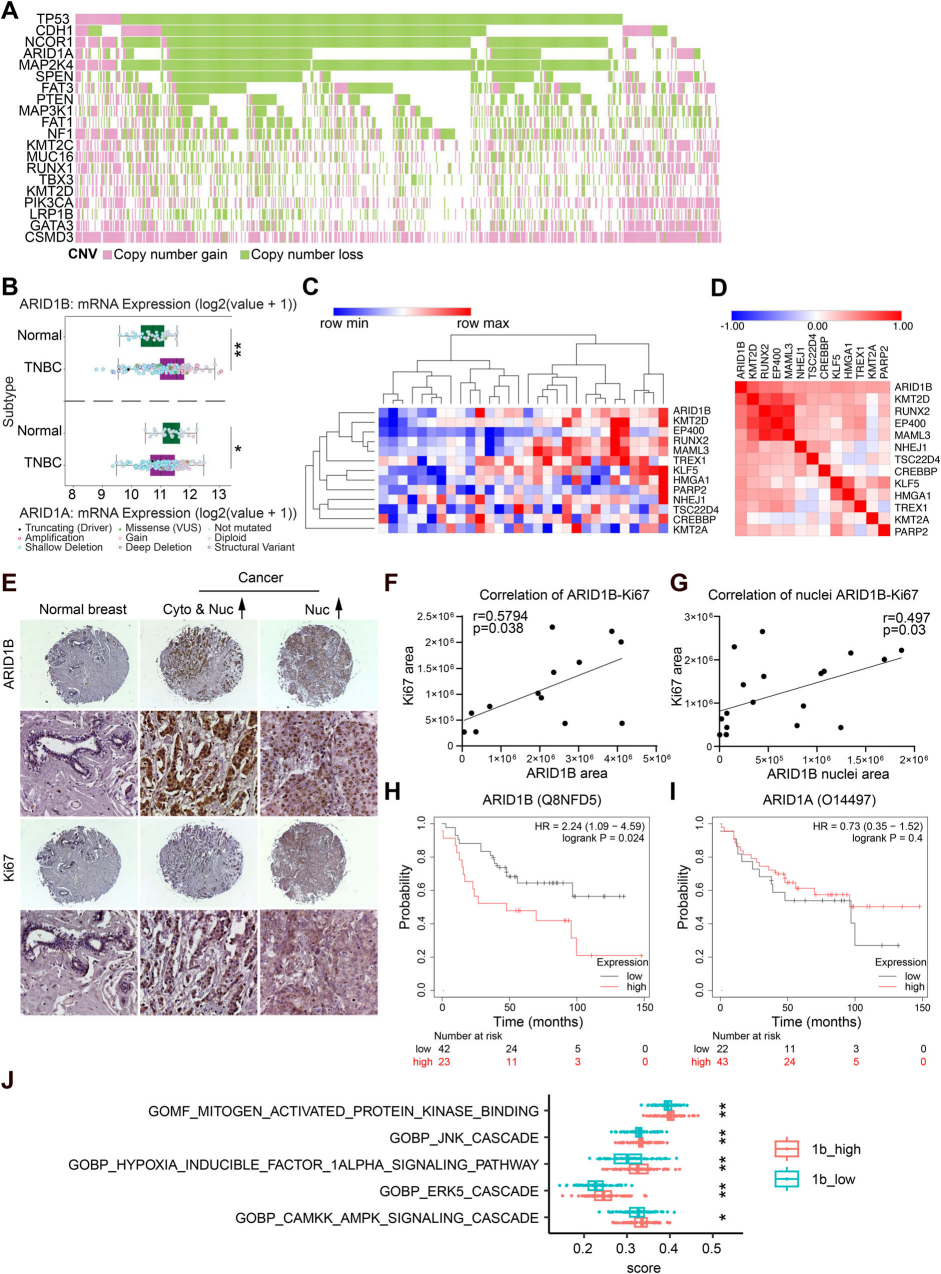
Elevated expression of ARID1B correlates with a poor prognosis of breast cancer. A) Genetic alterations in breast cancer were analyzed using the oncomatrix databases, and the frequencies of gene alterations were plotted. ARID1A is one of the most common and significant alterations observed in breast cancer. B) ARID1A and ARID1B mRNA expression levels were analyzed in human TNBC samples using the TCGA dataset. C) Heatmap and hierarchical clustering are based on the protein expression levels of the cancer cell proliferation‐ and stemness‐related proteins from 56 human TNBC samples. Each column represents a sample; each row represents a protein. The log 2 relative protein expression scale is depicted on the top left. D) Spearman's rank correlation analysis using the CPTAC TNBC dataset shows that ARID1B protein expression is highly positively correlated with cell proliferation‐ and stemness‐related proteins. E) 4X and 20X IHC of ARID1B and Ki67 in breast cancer and normal tissues. Cyto, cytoplasmic; Nuc, nuclear. F) Correlation between ARID1B and proliferative marker Ki67 in breast cancer. G) Correlation between nuclear ARID1B and proliferative marker Ki67 in breast cancer. H) Kaplan–Meier survival plot for breast cancer patients with ARID1B high‐ (*n* = 23) and low‐ (*n* = 42) expressing tumors. High protein levels of ARID1B are associated with worse prognosis in breast cancer patients. The Tang 2018 database and Kaplan–Meier Plotter were used for analysis.^[^ ^87^ ^]^ Time to follow‐up was measured up to 150 months. Logrank p‐value = 0.024. I) Kaplan–Meier survival plot for breast cancer patients with ARID1A high‐ (*n* = 43) and low‐ (n = 22) expressing tumors. Although the data were not statistically significant, high protein levels of ARID1A showed a trend of better survival in breast cancer patients, which didnot reach statistical significance. The Tang 2018 database and Kaplan–Meier Plotter were used for analysis.^[^ ^87^ ^]^ Time to follow‐up was measured up to 150 months. Logrank *p*‐value = 0.4. J) Comparison of ssGSEA enrichment scores for cell proliferation‐related pathways between patients with ARID1B low levels and ARID1B high levels. All *P*‐values are from Wilcoxon tests. *: *p* < 0.05; **: *p* < 0.01; ***: *p* < 0.001; ****: *p* < 0.0001.Elevated expression of ARID1B correlates with a poor prognosis of breast cancer. A) Genetic alterations in breast cancer were analyzed using the oncomatrix databases, and the frequencies of gene alterations were plotted. ARID1A is one of the most common and significant alterations observed in breast cancer. B) ARID1A and ARID1B mRNA expression levels were analyzed in human TNBC samples using the TCGA dataset. C) Heatmap and hierarchical clustering are based on the protein expression levels of the cancer cell proliferation‐ and stemness‐related proteins from 56 human TNBC samples. Each column represents a sample; each row represents a protein. The log 2 relative protein expression scale is depicted on the top left. D) Spearman's rank correlation analysis using the CPTAC TNBC dataset shows that ARID1B protein expression is highly positively correlated with cell proliferation‐ and stemness‐related proteins. E) 4X and 20X IHC of ARID1B and Ki67 in breast cancer and normal tissues. Cyto, cytoplasmic; Nuc, nuclear. F) Correlation between ARID1B and proliferative marker Ki67 in breast cancer. G) Correlation between nuclear ARID1B and proliferative marker Ki67 in breast cancer. H) Kaplan–Meier survival plot for breast cancer patients with ARID1B high‐ (*n* = 23) and low‐ (*n* = 42) expressing tumors. High protein levels of ARID1B are associated with worse prognosis in breast cancer patients. The Tang 2018 database and Kaplan–Meier Plotter were used for analysis.^[^ ^87^ ^]^ Time to follow‐up was measured up to 150 months. Logrank p‐value = 0.024. I) Kaplan–Meier survival plot for breast cancer patients with ARID1A high‐ (*n* = 43) and low‐ (n = 22) expressing tumors. Although the data were not statistically significant, high protein levels of ARID1A showed a trend of better survival in breast cancer patients, which didnot reach statistical significance. The Tang 2018 database and Kaplan–Meier Plotter were used for analysis.^[^ ^87^ ^]^ Time to follow‐up was measured up to 150 months. Logrank *p*‐value = 0.4. J) Comparison of ssGSEA enrichment scores for cell proliferation‐related pathways between patients with ARID1B low levels and ARID1B high levels. All *P*‐values are from Wilcoxon tests. *: *p* < 0.05; **: *p* < 0.01; ***: *p* < 0.001; ****: *p* < 0.0001.

Copy number alterations are a hallmark of cancer genomes, contributing to tumor development, progression, and therapeutic resistance. Among these, the ARID1A gene frequently exhibits copy number loss in breast cancer (Figure **1**A). ARID1A functions as a tumor suppressor, playing a critical role in maintaining chromatin architecture and regulating gene expression. Its loss is associated with disruption of chromatin remodeling and transcriptional regulation, driving tumorigenesis. Interestingly, emerging evidence suggests that ARID1B, the paralog of ARID1A, may play a compensatory role in maintaining chromatin integrity when ARID1A is lost. This potential upregulation of ARID1B raises important questions about its function in breast cancer progression and whether it acts as an adaptive mechanism or contributes to oncogenesis.

To understand the role of ARID1B in cancer development, we initially examined the gene expression profiles of ARID1B in pan‐cancer patients and analyzed its differential expression across various cancers. Using patient sample data obtained from the Clinical Proteomic Tumor Analysis Consortium (CPTAC), we compared ARID1B protein levels between normal and tumor tissues amongst ten different types of cancer, including breast cancer. Our analysis showed that ARID1B protein levels were significantly higher in breast cancer and glioblastoma compared to normal tissue (Figure S1A, Supporting Information). All other cancers showed either no significant difference in ARID1B expression between normal and tumor samples or a marked decrease in ARID1B expression in tumor tissues. The differential expression of ARID1B in pan‐cancer suggests that the role of ARID1B in cancer might be tissue‐specific. The marked increase in ARID1B expression in breast tumor tissues implies that ARID1B may be a promising therapeutic target for breast cancer. Moreover, the elevated ARID1B protein expression was detected in different molecular subtypes of breast cancer, including luminal, HER2+, and TNBC, compared to normal breast tissue (Figure S1B, Supporting Information). Analysis of mRNA expression levels reveals a significant downregulation of ARID1A in TNBC compared to normal tissue, which is consistent with its tumor suppressor role (Figure 1B). In contrast, ARID1B expression is markedly upregulated in TNBC, suggesting a potential compensatory mechanism or an oncogenic role (Figure 1B). To further investigate, we analyzed the protein expression levels of multiple SWI/SNF subunits in 56 human TNBC samples (Figure S1C, Supporting Information). ARID1A protein levels were consistently low across many individual samples, aligning with its frequent loss and tumor suppressor role in breast cancer. In contrast, ARID1B protein levels were significantly higher in the same samples, supporting the hypothesis that ARID1B may compensate for ARID1A loss or contribute to oncogenesis in TNBC. Moreover, there was a positive correlation between ARID1B protein expression and the expression of cell proliferation‐related proteins, as well as stemness‐related proteins (Figure 1C,D). These findings suggest that elevated ARID1B levels in TNBC may not only drive tumor growth but also enhance stemness characteristics, potentially contributing to therapy resistance and tumor recurrence.

Additionally, immunohistochemistry (IHC) analysis of breast cancer patient specimens confirmed the high levels of ARID1B in cancerous tissues (Figure 1E). In normal tissues, only occasional cells exhibited nuclear expression of ARID1B, whereas in cancerous tissues, most nuclei showed positive staining for ARID1B, indicating an overall increase in nuclear levels. Interestingly, some breast cancer samples also displayed a strong ARID1B signal in the cytoplasm, while others did not, suggesting dysregulation of ARID1B and its nuclear import mechanisms. IHC analysis of ARID1A levels in breast cancer samples showed a negative correlation between ARID1B and ARID1A (Figure S1D,E). Moreover, we observed a statistically significant correlation between high nuclear ARID1B expression and increased levels of Ki67, a marker of cell proliferation (Figure 1E–G).

To assess the impact of ARID1B levels on overall patient survival, we conducted a cumulative survival analysis. Consistent with previous reports,^[^ ^25^, ^26^ ^]^ we observed that high mRNA and protein levels of ARID1B are associated with a worse breast cancer prognosis (Figure 1H, Figure S1F, Supporting Information). Given that ARID1A and ARID1B are mutually exclusive in the BAF complex, we also analyzed the impact of ARID1A expression on overall patient survival (Figure 1I, Figure S1G, Supporting Information). When comparing patients with low and high ARID1A expression, we found that the overall survival period for patients with high ARID1A expression was longer (Figure 1I). Although the difference in survival periods was not significant, the general trend supports previous findings that ARID1A functions as a tumor suppressor, playing an opposite role to that of its mutually exclusive counterpart ARID1B.^[^ ^28^, ^29^, ^30^ ^]^ Further analysis of The Cancer Genome Atlas (TCGA) data showed that the promoter methylation levels of ARID1B in breast cancer are significantly lower than those in normal tissue (Figure S1H, Supporting Information), while the promoter methylation levels of ARID1A in breast cancer are significantly higher than those in normal tissue (Figure S1I, Supporting Information).

Additionally, we performed single‐sample gene set enrichment analysis (ssGSEA) to evaluate enrichment scores for cell proliferation‐related pathways and cancer progenitor cell‐related pathways in breast cancer patients grouped by ARID1B expression levels. Patients with high ARID1B expression exhibited significantly higher enrichment scores for both cell proliferation and cancer progenitor cell‐related pathways compared to those with low ARID1B expression (Figure 1J, Figure S1J, Supporting Information).

Given the mutually exclusive roles of ARID1A and ARID1B within the BAF chromatin remodeling complex, we hypothesized that ARID1B‐dependent transcriptional programs might be particularly enhanced in the context of ARID1A mutations. To test this hypothesis, we conducted bioinformatic analyses comparing transcriptional profiles of ARID1B‐high versus ARID1B‐low breast cancer samples. This analysis identified 397 significantly upregulated and 596 significantly downregulated genes, classified as ARID1B‐dependent transcriptome (Figure S2A, Supporting Information).

Next, we examined differentially expressed genes (DEGs) between ARID1A‐mutant and ARID1A‐wildtype (WT) breast cancer samples to understand the transcriptional interplay between ARID1A and ARID1B. Hierarchical clustering showed that ARID1B‐dependent transcriptional changes were more pronounced in ARID1A‐mutant samples, with ARID1B‐upregulated genes exhibiting higher expression and ARID1B‐downregulated genes exhibiting lower expression specifically in the context of ARID1A mutation, suggesting a potential transcriptional association between ARID1B activity and ARID1A mutation status (Figure S2B,C, Supporting Information).

We identified 49 genes (10.6%) concurrently upregulated and 168 genes (20.1%) concurrently downregulated in ARID1A‐mutant and ARID1B‐high breast cancer samples (Figure S2D,E, Supporting Information). Subsequent clustering analysis focusing specifically on these overlapping genes revealed more distinct clustering patterns, clearly demonstrating enhanced transcriptional changes associated with ARID1A‐mutant samples (Figure S2F,G, Supporting Information). These data collectively support the hypothesis that ARID1B activity is functionally amplified specifically in ARID1A‐mutant breast cancers, highlighting a dominant oncogenic role of ARID1B‐dependent transcriptional programs in this context.

Additionally, we evaluated the functional impact of ARID1B KO in ARID1A‐mutant T47D and ARID1A KO MDA‐MB‐468 cells (Figure S3). In ARID1A‐mutant T47D cells, ARID1B KO significantly reduced cell growth and colony formation, suggesting that ARID1B is essential for cell survival and growth specifically in the context of ARID1A mutants (Figure S3A,B, Supporting Information). In MDA‐MB‐468 cells, loss of ARID1A significantly promoted cell growth and colony formation, consistent with the known tumor‐suppressive role of ARID1A (Figure S3C,D, Supporting Information). Conversely, ARID1B KO significantly reduced cell growth and colony formation, supporting its pro‐tumorigenic function (Figure S3C,D, Supporting Information). Depletion of both ARID1B and ARID1A resulted in a growth phenotype comparable with ARID1B KO alone, indicating that ARID1B loss overrides the growth‐promoting phenotype driven by ARID1A KO. This suggests that ARID1B activity may be essential for tumor growth in the context of ARID1A mutation or downregulation.

Taken together, our findings highlight that the aberrant accumulation of ARID1B, which could be due to lower promoter methylation, significantly correlates with a poor prognosis in breast cancer patients.^[^ ^25^, ^26^ ^]^ Our findings suggest that elevated ARID1B levels in TNBC not only drive tumor growth through enhanced cell proliferation but also promote stem cell‐like characteristics associated with cancer progression and therapy resistance. Together, this highlights the pivotal role of ARID1B in TNBC and its potential as a therapeutic target.

### 2.2 Elevated Expression of ARID1B Promotes Tumorigenesis and Dampens Therapeutic Efficacy

**Figure 2:**
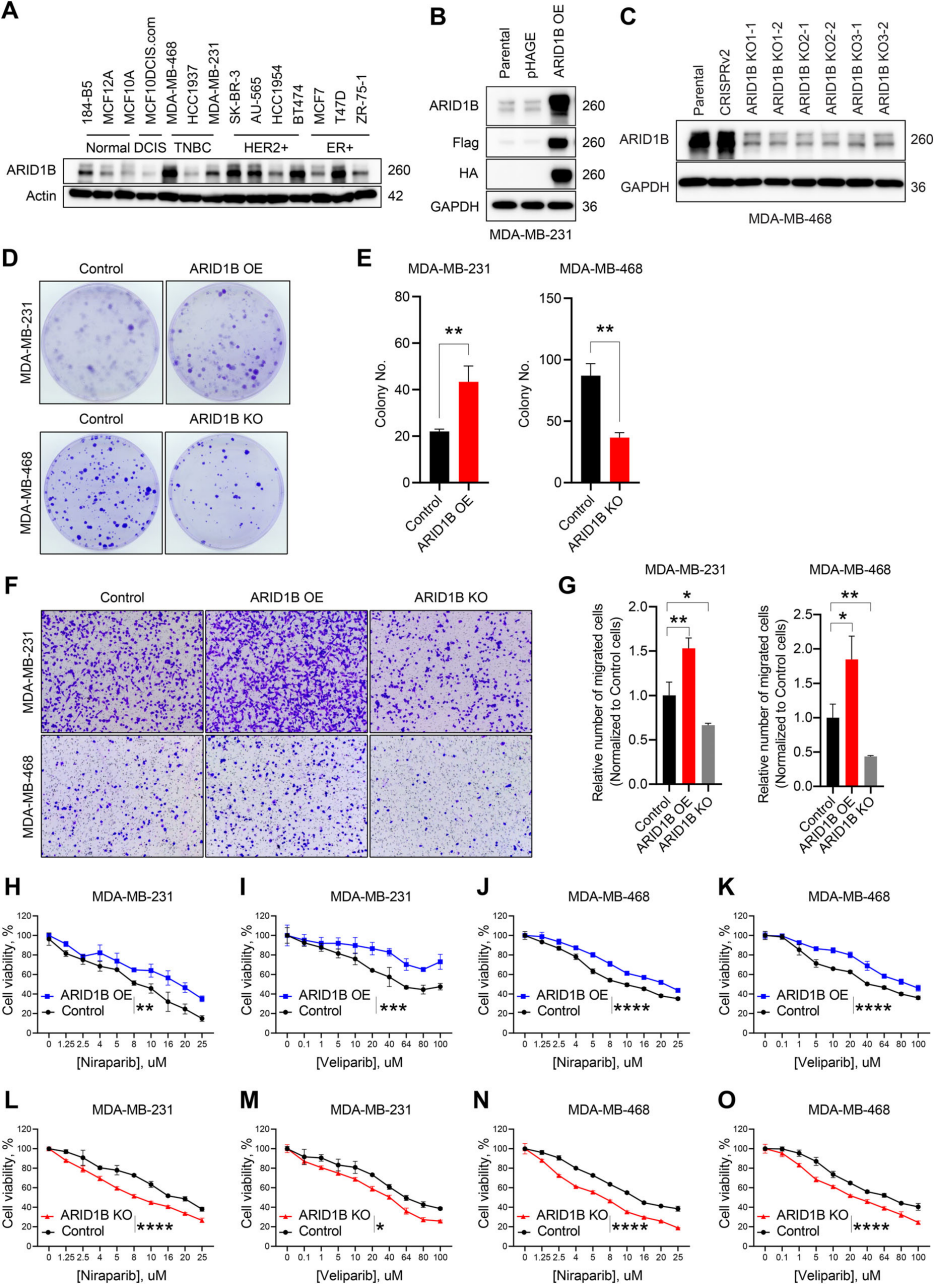
Elevated expression of ARID1B promotes tumorigenesis and dampens therapeutic efficacy. A) Immunoblot analysis of ARID1B in normal breast epithelial cells (184‐B5), in non‐tumorigenic breast epithelial cell lines (MCF12A and MCF10A), ductal carcinoma in situ (MCF10DCIS.com), as well as in different breast cancer cell lines, including TNBC (MDA‐MB‐468, HCC1937, MDA‐MB‐231), HER2+ (SK‐BR‐3, AU‐565, HCC1954, BT474), and ER+ (MCF7, T47D, ZR‐75‐1). ARID1B is elevated in breast cancer cells compared to normal and non‐tumorigenic breast epithelial cells. Actin was used as a loading control. B) Immunoblot analysis of ARID1B, Flag, and HA levels inWT (Parental), empty vector control (Control), and ARID1B OE MDA‐MB‐231 cell lines. GAPDH was used as a loading control. C) Immunoblot analysis of ARID1B in WT (Parental), empty vector control (CRISPRv2), and ARID1B KO MDA‐MB‐468 cells. ARID1B KO 1–3 cell lines were each generated using different sgRNAs. GAPDH was used as a loading control. D) Representative images of colony formation assay in response to OE and KO of ARID1B in breast cancer cells. E) Quantification of colony numbers formed in ARID1B OE MDA‐MB‐231 and ARID1B KO MDA‐MB‐468 cells compared to their corresponding empty vector controls. F) Effect of ARID1B on cell migration in MDA‐MB‐231 and MDA‐MB‐468 cells compared to control. Representative images from cell migration assays comparing ARID1B OE and ARID1B KO to control cells are shown. G) Quantification of cell migration in MDA‐MB‐231 and MDA‐MB‐468 breast cancer cells in response to ARID1B OE and ARID1B KO compared to control. ARID1B OE cells exhibited a significantly increased migration compared to control cells, whereas ARID1B KO markedly reduced cell migration. H,I) Dose‐response curves for PARP inhibitors Niraparib H) and Veliparib I) after 72 h of treatment in MDA‐MB‐231 cells comparing empty vector control (Control) and ARID1B OE cells. Statistical significance was determined by two‐way ANOVA. J,K) Dose‐response curves for Niraparib J) and Veliparib K) after 72 h of treatment in MDA‐MB‐468 cells comparing empty vector control (Control) and ARID1B OE cells. Statistical significance was determined by two‐way ANOVA. L,M) Dose‐response curves for Niraparib (L) and Veliparib (M) after 72 h of treatment in MDA‐MB‐231 cells comparing empty vector control (Control) and ARID1B KO cells. Statistical significance was determined by two‐way ANOVA. N,O) Dose‐response curves for Niraparib (N) and Veliparib (O) after 72 h of treatment in MDA‐MB‐468 cells comparing empty vector control (Control) and ARID1B KO cells. Statistical significance was determined by two‐way ANOVA. *P*‐values are summarized with asterisks: * – *p* ≤ 0.05, ** – *p* ≤ 0.01, *** – *p* ≤ 0.001, **** – *p* ≤ 0.0001.Elevated expression of ARID1B promotes tumorigenesis and dampens therapeutic efficacy. A) Immunoblot analysis of ARID1B in normal breast epithelial cells (184‐B5), in non‐tumorigenic breast epithelial cell lines (MCF12A and MCF10A), ductal carcinoma in situ (MCF10DCIS.com), as well as in different breast cancer cell lines, including TNBC (MDA‐MB‐468, HCC1937, MDA‐MB‐231), HER2+ (SK‐BR‐3, AU‐565, HCC1954, BT474), and ER+ (MCF7, T47D, ZR‐75‐1). ARID1B is elevated in breast cancer cells compared to normal and non‐tumorigenic breast epithelial cells. Actin was used as a loading control. B) Immunoblot analysis of ARID1B, Flag, and HA levels inWT (Parental), empty vector control (Control), and ARID1B OE MDA‐MB‐231 cell lines. GAPDH was used as a loading control. C) Immunoblot analysis of ARID1B in WT (Parental), empty vector control (CRISPRv2), and ARID1B KO MDA‐MB‐468 cells. ARID1B KO 1–3 cell lines were each generated using different sgRNAs. GAPDH was used as a loading control. D) Representative images of colony formation assay in response to OE and KO of ARID1B in breast cancer cells. E) Quantification of colony numbers formed in ARID1B OE MDA‐MB‐231 and ARID1B KO MDA‐MB‐468 cells compared to their corresponding empty vector controls. F) Effect of ARID1B on cell migration in MDA‐MB‐231 and MDA‐MB‐468 cells compared to control. Representative images from cell migration assays comparing ARID1B OE and ARID1B KO to control cells are shown. G) Quantification of cell migration in MDA‐MB‐231 and MDA‐MB‐468 breast cancer cells in response to ARID1B OE and ARID1B KO compared to control. ARID1B OE cells exhibited a significantly increased migration compared to control cells, whereas ARID1B KO markedly reduced cell migration. H,I) Dose‐response curves for PARP inhibitors Niraparib H) and Veliparib I) after 72 h of treatment in MDA‐MB‐231 cells comparing empty vector control (Control) and ARID1B OE cells. Statistical significance was determined by two‐way ANOVA. J,K) Dose‐response curves for Niraparib J) and Veliparib K) after 72 h of treatment in MDA‐MB‐468 cells comparing empty vector control (Control) and ARID1B OE cells. Statistical significance was determined by two‐way ANOVA. L,M) Dose‐response curves for Niraparib (L) and Veliparib (M) after 72 h of treatment in MDA‐MB‐231 cells comparing empty vector control (Control) and ARID1B KO cells. Statistical significance was determined by two‐way ANOVA. N,O) Dose‐response curves for Niraparib (N) and Veliparib (O) after 72 h of treatment in MDA‐MB‐468 cells comparing empty vector control (Control) and ARID1B KO cells. Statistical significance was determined by two‐way ANOVA. *P*‐values are summarized with asterisks: * – *p* ≤ 0.05, ** – *p* ≤ 0.01, *** – *p* ≤ 0.001, **** – *p* ≤ 0.0001.

To decipher the impact of ARID1B cellular protein abundance in tumor malignancy, we examined the expression of ARID1B in a panel of breast cancer cell lines, including mammary gland epithelial, luminal, HER2+ and TNBC. As shown in Figure **2**A, we observed a significant upregulation of ARID1B in all types of breast cancer cells when compared to the normal breast epithelial (184‐B5) or non‐tumorigenic breast epithelial cell lines (MCF10A and MCF12A). The accumulation of ARID1B in aggressive breast cancer subtypes such as TNBC attracts our attention because of its aggressive malignant feature with a lack of suitable therapeutic options. To further determine the relevance of aberrant accumulation in tumor development, we either overexpressed (OE) or depleted (KO) ARID1B in breast cancer cells and evaluated its impact on cancer cell growth (Figure 2B,C, Figure S4A, Supporting Information). A colony‐formation assay confirmed that the formation of colonies was increased in ARID1B OE cells and decreased in ARID1B KO cells (Figure 2D,E, Figure S4B,C, Supporting Information). Moreover, a cell migration assay demonstrated that upregulation of ARID1B significantly enhanced cancer cell migration, whereas ARID1B downregulation substantially reduced it (Figure 2F,G).

We next investigated whether ARID1B accumulation in breast cancer affects the efficacy of current targeted therapy regimens. Specifically, we examined how elevated ARID1B expression influences the survival of TNBC and HER2+ cells in response to different types of therapeutics, including PARP inhibitors (Niraparib and Veliparib), tyrosine kinase inhibitors (Tucatinib and Neratinib), PI3K inhibitors (BYL719 and GDC0914), and topoisomerase I inhibitor (SN38) (Figure 2H–K, Figures S4D–F,S5 Supporting Information). In TNBC cells, elevated ARID1B expression significantly decreased sensitivity to the PARP inhibitors Niraparib and Veliparib, as demonstrated by dose‐response curves and IC50 shifts in both MDA‐MB‐231 and MDA‐MB‐468 cells (Figure 2H–K, Figure S5, Supporting Information). Conversely, ARID1B KO significantly increased sensitivity to these agents in both cell lines (Figure 2L‐O, S5). These results demonstrate that ARID1B promotes resistance to PARP inhibition across multiple TNBC models, as shown through both gain‐ and loss‐of‐function approaches. Similarly, in HER2+ cells, ARID1B OE diminished responsiveness to HER2‐targeted agents Tucatinib and Neratinib (Figure S6A–E, Supporting Information), while ARID1B KO enhanced sensitivity to these therapies (Figure S6, Supporting Information). Notably, ARID1B OE did not affect the sensitivity to PI3K inhibitors or SN38 (Figure S4D–F, Supporting Information). Together, these results suggest that elevated ARID1B expression promotes tumorigenesis and exerts a context‐dependent effect on drug response.

Since ARID1B protein expression positively correlates with stemness‐related proteins in breast cancer patients (Figure 1C,D), we further investigated the role of ARID1B OE in the cancer stem cell maintenance using 3D mammosphere formation assay. ARID1B OE significantly increased the number of spheroids (Figure S7, Supporting Information) and elevated the portion of CD44+/CD24‐ and ALDH‐positive cancer stem cell populations (Figure S7, Supporting Information), suggesting that ARID1B plays a critical role in promoting cell survival and the maintenance of cancer stem cells.

### 2.3 Nuclear Accumulation of ARID1B Results in a Reduction of ARID1A‐Bound BAF Complexes

**Figure 3:**
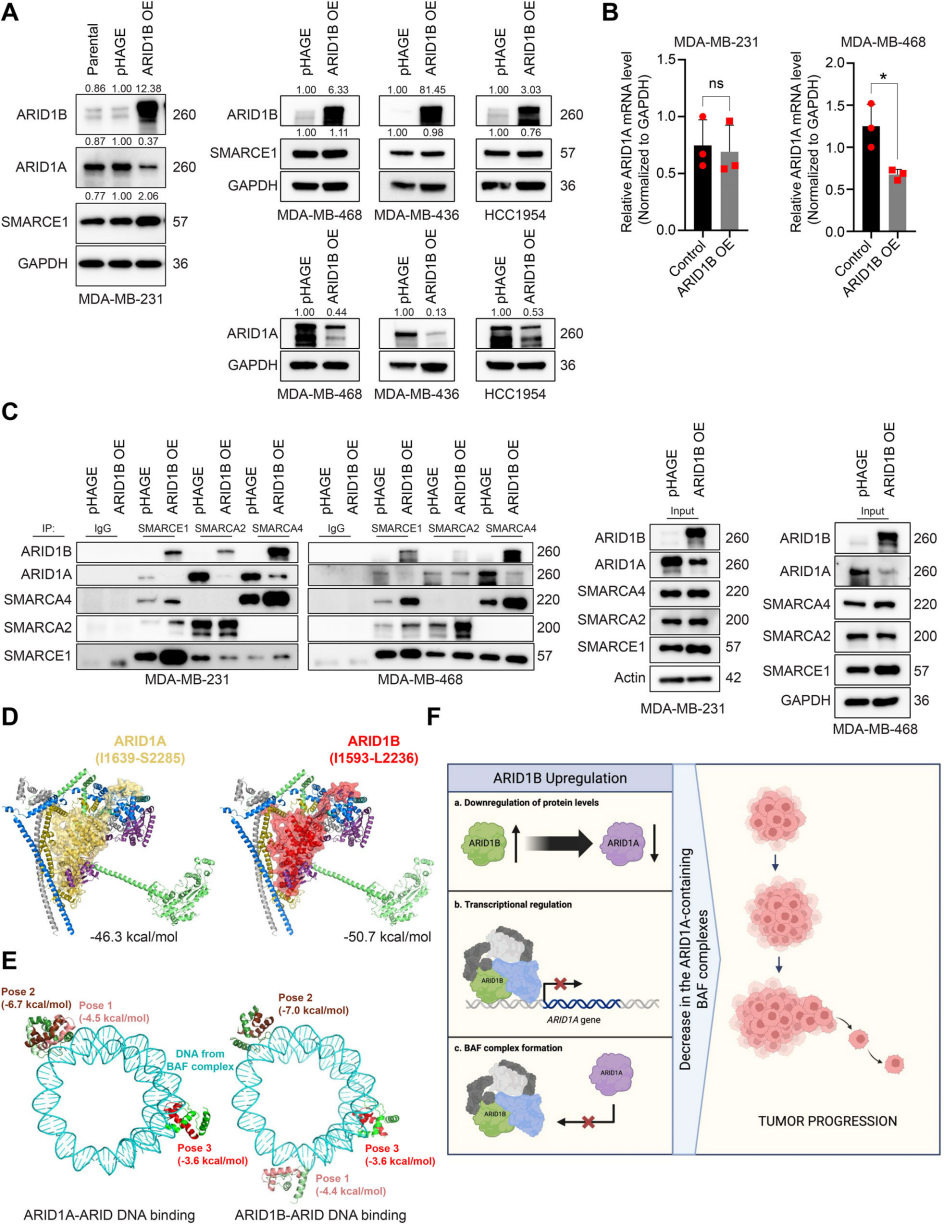
Nuclear accumulation of ARID1B results in a reduction of ARID1A‐bound BAF complexes. A) Immunoblot analysis of ARID1A and SMARCE1 levels in WT (Parental), empty vector control (pHAGE), and ARID1B OE breast cell lines. The levels of ARID1A and SMARCE1 were analyzed in MDA‐MB‐231, MDA‐MB‐468, MDA‐MB‐436, and HCC1954 cell lines. GAPDH was used as a loading control. B) mRNA levels of ARID1A in empty vector control and ARID1B OE MDA‐MB‐231 and ‐468 cell lines. The data are presented as mean ± SD; n = 3 independent experiments, two‐tailed t‐tests. C) Immunoblot analysis of total cell lysate protein input and immunoprecipitates (IP) by SMARCE1, SMARCA2, and SMARCA4‐specific antibodies from MDA‐MB‐231 and MDA‐MB‐468 empty vector control (pHAGE) and ARID1B OE cells. Immunoglobulin G (IgG) IPs were used as negative control. D) Comparison of the complex formed by the BAF core with ARID1A (*left*) and ARID1B (*right*). E) Results from docking simulations of the ARID of ARID1A (*left*) and ARID1B (*right*) onto the nucleosome within the BAF complex. The three most energetically favorable poses are shown in each case, along with their corresponding binding energies. The HTH motifs near AT‐rich motifs are shown in *salmon* and *brown* (***poses 1 and 2***), and the HTH motif in the binding of ARID to solvent‐accessible DNA is shown in *red*. F) Schematic description of the mechanisms by which ARID1B OE suppresses ARID1A expression and reduces the formation of the ARID1A‐bound BAF complexes, leading to tumor growth and progression. Created with BioRender.com.Nuclear accumulation of ARID1B results in a reduction of ARID1A‐bound BAF complexes. A) Immunoblot analysis of ARID1A and SMARCE1 levels in WT (Parental), empty vector control (pHAGE), and ARID1B OE breast cell lines. The levels of ARID1A and SMARCE1 were analyzed in MDA‐MB‐231, MDA‐MB‐468, MDA‐MB‐436, and HCC1954 cell lines. GAPDH was used as a loading control. B) mRNA levels of ARID1A in empty vector control and ARID1B OE MDA‐MB‐231 and ‐468 cell lines. The data are presented as mean ± SD; n = 3 independent experiments, two‐tailed t‐tests. C) Immunoblot analysis of total cell lysate protein input and immunoprecipitates (IP) by SMARCE1, SMARCA2, and SMARCA4‐specific antibodies from MDA‐MB‐231 and MDA‐MB‐468 empty vector control (pHAGE) and ARID1B OE cells. Immunoglobulin G (IgG) IPs were used as negative control. D) Comparison of the complex formed by the BAF core with ARID1A (*left*) and ARID1B (*right*). E) Results from docking simulations of the ARID of ARID1A (*left*) and ARID1B (*right*) onto the nucleosome within the BAF complex. The three most energetically favorable poses are shown in each case, along with their corresponding binding energies. The HTH motifs near AT‐rich motifs are shown in *salmon* and *brown* (***poses 1 and 2***), and the HTH motif in the binding of ARID to solvent‐accessible DNA is shown in *red*. F) Schematic description of the mechanisms by which ARID1B OE suppresses ARID1A expression and reduces the formation of the ARID1A‐bound BAF complexes, leading to tumor growth and progression. Created with BioRender.com.

ARID1B and ARID1A share 49.4% sequence identity, and, as such, their overall structure is highly similar. They are mutually exclusive subunits of the BAF complex (i.e., the BAF chromatin remodeling complexes can contain either ARID1A or ARID1B, but not both).^[^ ^11^, ^13^, ^16^ ^]^ It has been previously reported that ARID1A‐ or ARID1B‐containing BAF complexes play opposing roles in regulating cell proliferation and osteoblast differentiation.^[^ ^13^, ^15^, ^16^, ^31^, ^32^ ^]^ Given this notion, we sought to determine whether OE of ARID1B has any impact on the composition of the BAF complex in breast cancer cells. As shown in **Figure** **3**A,B and Figure S8A (Supporting Information), elevated expression of ARID1B significantly decreased ARID1A levels in multiple breast cancer cell lines at both the protein and mRNA levels. In contrast, KO of ARID1B resulted in a significant increase in ARID1A levels and KO of ARID1A resulted in a significant increase in ARID1B levels (Figure S8B,C, Supporting Information). Moreover, among the tested ER+ breast cancer cells, ARID1A mutant T47D cells had higher ARID1B mRNA and protein levels compared to those of ARID1A WT cells (Figure S8D,E). Taken together, these data suggest a reciprocal negative regulatory relationship between ARID1A and ARID1B, which further supported the negative regulation of ARID1A by ARID1B.

As shown in Figure 3B, ARID1B OE led to a reduction in ARID1A mRNA levels in MDA‐MB‐468 cells, but not in MDA‐MB‐231 cells. Interestingly, despite unchanged ARID1A mRNA levels in MDA‐MB‐231 cells, ARID1A protein levels were still markedly reduced in the ARID1B OE cells. To explore whether this reduction was due to enhanced protein degradation, we performed a cycloheximide (CHX) chase assay to measure protein stability. Our results showed that ARID1A protein levels decline more rapidly in ARID1B OE cells compared to control cells, indicating that ARID1B OE accelerates ARID1A turnover and reduces its half‐life. In contrast, ARID1B KO led to increased stability of the ARID1A protein compared to control (Figure S8F–I, Supporting Information).

To further investigate whether ARID1B upregulation alters the composition of the BAF complex itself, we next performed immunoprecipitation (IP) assays to examine the composition of BAF complexes upon ARID1B OE. Using antibodies against SMARCE1, a core subunit shared by both ARID1A‐ and ARID1B‐containing BAF complexes (Figure 3C), we found that ARID1B OE led to an increase in ARID1B‐SMARCA2/SMARCA4‐containing BAF complexes, accompanied by a reduction in ARID1A‐containing BAF complexes (Figure 3C). These findings support a model in which ARID1B displaces ARID1A from the complex, potentially through competitive binding. Similar results were observed when BAF complexes were immunoprecipitated using antibodies against the mutually exclusive ATPases subunits SMARCA2 and SMARCA4 (Figure 3C).

To further understand the BAF complex assembly and whether ARID1A and ARID1B compete for binding to the BAF core, we modeled their interactions in silico using the structure of nucleosome‐bound human ARID1A‐containing BAF complex (Figure S8J, Supporting Information)^[^ ^33^ ^]^ (PDB: 6ltj) and the structural model predicted by AlphaFold2^[^ ^34^, ^35^, ^36^ ^]^ for ARID1B. The latter was structurally aligned against the ARID1A structure within the BAF complex to construct a BAF complex with ARID1B. The C‐terminal region spanning residues I1593‐L2236 of ARID1B aligned with the I1639‐S2285 region of ARID1A in the BAF complex. Residues within the region of ARID1B that corresponded to their unresolved counterpart in ARID1A were removed from the superimposed ARID1B BAF complex. A significant portion of ARID1B, particularly the N‐terminal region, was predicted to be largely disordered by AlphaFold2. However, in the newly generated BAF complex, we only included regions that had an average predicted Local Distance Difference Test (pLDDT) score of 90.88, indicating a high‐confidence prediction. The superimposed ARID1B‐bound BAF complex was further refined using the HADDOCK interface.^[^ ^37^ ^]^ The resulting complex with ARID1B is shown in Figure 3D *(right)* in comparison to the complex with ARID1A *(left)*.

Next, we used the PRODIGY web server^[^ ^38^ ^]^ to predict the binding affinity of ARID1B with the BAF core and compared it to that of ARID1A. As shown in Figure 3D, PRODIGY predictions indicate that ARID1B has a stronger binding affinity (−50.7 kcal mol^−1^) compared to ARID1A (−46.3 kcal mol^−1^), suggesting that ARID1B is more likely to interact with the BAF core when both proteins are equally expressed or equally accessible to the BAF complex. However, biochemical validation is necessary to confirm this prediction.

We further compared the DNA‐binding poses of the ARID DNA‐binding domain within the subunits ARID1A and ARID1B. ARID is a conserved domain characterized by a helix‐turn‐helix (HTH) motif. The ARIDs of ARID1A and ARID1B have been described to bind DNA non‐specifically and with inconsistent affinities.^[^ ^39^ ^]^ We adopted three strategies for docking the ARIDs from ARID1A and ARID1B onto the DNA structure resolved in the BAF complex. First, we carried out guided docking simulations using HADDOCK,^[^ ^40^ ^]^ focusing on the binding of the HTH motif onto AT‐rich DNA sites and solvent‐accessible DNA. Figure 3E *(left)* shows three docked poses of the ARID1A ARID, selected from amongst 96 alternative poses as those with the strongest interaction energies (evaluated by FoldX^[^ ^41^ ^]^). Figure 3E *(right)* shows the counterpart for ARID1B ARID. The similar interaction energy of the ARID domains bound to the most favorable site (*pose 2*), as well as other less favorable sites, suggests ARID1A and ARID1B have a comparable affinity to bind DNA (Figure 3E). Yet, as predicted by PRODIGY, ARID1B may have a higher binding affinity for the BAF core than ARID1A in structural models, suggesting that ARID1B is more likely to interact with DNA in a competitive binding scenario.

Together, these data suggest that elevated expression of ARID1B negatively regulates ARID1A and leads to a decrease in the number of ARID1A‐containing BAF complexes (Figure 3F). A shift from ARID1A‐ to ARID1B‐bound BAF complexes can result in a switch from tumor‐suppression to oncogenesis action in SWI/SNF chromatin remodelers.

### 2.4 Interplay Between ARID1B and ARID1A in Regulating Gene Expression

**Figure 4:**
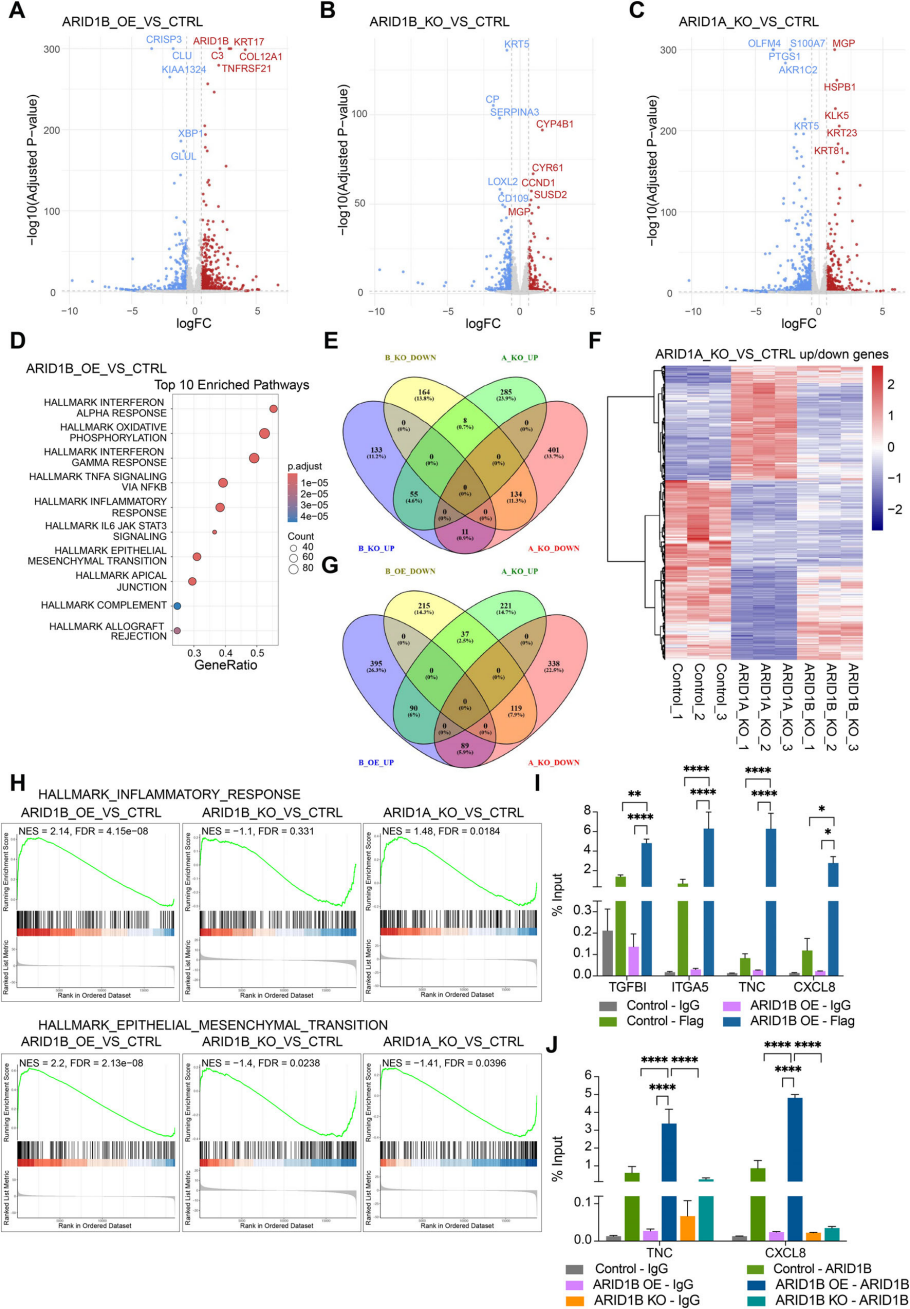
Interplay between ARID1B and ARID1A in regulating gene expression. A–C) Volcano plots showing DEGs in ARID1B OE A), ARID1B KO B), and ARID1A KO C) MDA‐MB‐468 cells compared to the control. D) Hallmark pathway enrichment analysis of ARID1B OE versus Control cells. E) Venn diagram represents overlapping upregulated and downregulated genes between ARID1B KO and ARID1A KO MDA‐MB‐468 cells. F) Heatmap showing the expression of ARID1A‐dependent genes in ARID1A KO and ARID1B KO MDA‐MB‐468 cells. G) Venn diagram represents overlapping upregulated and downregulated genes between ARID1B OE and ARID1A KO MDA‐MB‐468 cells. H) GSEA of inflammatory response and EMT hallmark pathways in ARID1B OE, ARID1B KO, and ARID1A KO MDA‐MB‐468 cells. I) ChIP‐qPCR analysis confirms ectopic ARID1B binding at the *TGFBI*, *ITGA5*, *TNC*, and *CXCL8* promoters in ARID1B OE MDA‐MB‐468 cells. Flag and IgG ChIP were performed in ARID1B OE and control cells. J) ChIP‐qPCR analysis confirms endogenous ARID1B binding at the *TNC* and *CXCL8* promoters in ARID1B OE MDA‐MB‐468 cells. ARID1B and IgG ChIP were performed in ARID1B OE, ARID1B KO, and control cells. I, J) Data are represented as mean±SEM of three independent experiments. Statistical significance was determined by two‐way ANOVA followed by Tukey's multiple comparison test. *P*‐values are summarized as: **** – *p* < 0.0001.Interplay between ARID1B and ARID1A in regulating gene expression. A–C) Volcano plots showing DEGs in ARID1B OE A), ARID1B KO B), and ARID1A KO C) MDA‐MB‐468 cells compared to the control. D) Hallmark pathway enrichment analysis of ARID1B OE versus Control cells. E) Venn diagram represents overlapping upregulated and downregulated genes between ARID1B KO and ARID1A KO MDA‐MB‐468 cells. F) Heatmap showing the expression of ARID1A‐dependent genes in ARID1A KO and ARID1B KO MDA‐MB‐468 cells. G) Venn diagram represents overlapping upregulated and downregulated genes between ARID1B OE and ARID1A KO MDA‐MB‐468 cells. H) GSEA of inflammatory response and EMT hallmark pathways in ARID1B OE, ARID1B KO, and ARID1A KO MDA‐MB‐468 cells. I) ChIP‐qPCR analysis confirms ectopic ARID1B binding at the *TGFBI*, *ITGA5*, *TNC*, and *CXCL8* promoters in ARID1B OE MDA‐MB‐468 cells. Flag and IgG ChIP were performed in ARID1B OE and control cells. J) ChIP‐qPCR analysis confirms endogenous ARID1B binding at the *TNC* and *CXCL8* promoters in ARID1B OE MDA‐MB‐468 cells. ARID1B and IgG ChIP were performed in ARID1B OE, ARID1B KO, and control cells. I, J) Data are represented as mean±SEM of three independent experiments. Statistical significance was determined by two‐way ANOVA followed by Tukey's multiple comparison test. *P*‐values are summarized as: **** – *p* < 0.0001.

To further understand the interplay between ARID1B and ARID1A and to investigate the mechanisms by which ARID1B promotes tumorigenesis in TNBC, we performed RNA‐seq analysis in ARID1B OE, ARID1B KO, ARID1A KO, and control MDA‐MB‐468 cells. RNA‐seq analysis revealed 574 upregulated and 371 downregulated genes in response to ARID1B OE, 199 upregulated and 306 downregulated genes in response to ARID1B KO, and 348 upregulated and 546 downregulated genes in response to ARID1A KO (**Figure** **4**A–C, Figure S9A, Supporting Information).

Pathway enrichment analysis revealed that genes upregulated by ARID1B OE were significantly enriched in pathways frequently linked to oncogenic processes in breast cancer and other malignancies, including epithelial–mesenchymal transition (EMT), TNFα signaling via NFκB, inflammatory response, interferon alpha response, interferon gamma response, and IL6/JAK/STAT3 signaling (Figure 4D). In contrast, ARID1B KO led to significant downregulation of genes associated with several oncogenic pathways, such as mitotic spindle, androgen response, heme metabolism, UV response (DN), mTORC1 signaling, hypoxia, and glycolysis (Figure S9B, Supporting Information). These transcriptional changes are consistent with a suppression of tumor growth upon ARID1B downregulation.

Additionally, pathway enrichment analysis of ARID1A KO cells revealed significant upregulation of genes involved in cell cycle progression, including the G2/M checkpoint, E2F targets, and mitotic spindle, while genes enriched in glycolysis, hypoxia, mTORC1 signaling, oxidative phosphorylation, fatty acid metabolism, MYC targets v1, and adipogenesis were downregulated (Figure S9C, Supporting Information). These transcriptional changes suggest that ARID1A normally restrains cell proliferation while supporting other metabolic or stress response pathways, consistent with its tumor‐suppressive function.

To understand the differences between the ARID1A‐ and ARID1B‐dependent transcriptomes, we performed a comparative analysis of ARID1A KO and ARID1B KO cells. The analysis revealed that 134 genes were downregulated and 55 genes were upregulated in both ARID1A KO and ARID1B KO conditions (Figure 4E). Although some target genes are shared, ARID1A KO and ARID1B KO each resulted in a substantial number of unique transcriptional changes (Figure 4E). In ARID1B KO cells, 133 unique genes were upregulated and 164 unique genes were downregulated, while ARID1A KO cells exhibited 285 uniquely upregulated genes and 401 uniquely downregulated genes, which were enriched in pathways including hypoxia, glycolysis, EMT, estrogen response, inflammatory response, TNFα signaling via NFκB, KRAS signaling (Up), p53 pathway, and angiogenesis (Figure 4E, Figure S9D, Supporting Information).

Hierarchical clustering analysis of the ARID1A‐dependent transcriptome comparing ARID1A KO and ARID1B KO conditions revealed a major cluster of genes that were downregulated upon ARID1A KO and similarly downregulated in ARID1B KO cells, whereas a separate cluster remained unaffected by ARID1B KO (Figure 4F). Among the genes upregulated by ARID1A KO, a subset of genes was also upregulated in ARID1B KO cells, suggesting that ARID1A and ARID1B co‐regulate a substantial portion of the ARID1A‐dependent transcriptional program, particularly with respect to gene activation.

Since ARID1B OE results in downregulation of ARID1A, and its pro‐tumorigenic effects may be mediated in part through this mechanism, we conducted a comparative analysis of ARID1B OE and ARID1A KO conditions (Figure 4G). This analysis revealed that 119 genes were downregulated and 90 genes were upregulated in both ARID1A KO and ARID1B OE conditions. Pathway enrichment analysis of these overlapping genes showed involvement in hypoxia, EMT, xenobiotic metabolism, inflammatory response, and the p53 pathway (Figure S10A, Supporting Information).

In addition, ARID1B OE regulated a substantial number of unique genes: 395 genes were upregulated and 215 genes were downregulated exclusively in the ARID1B OE condition (Figure 4G). These unique genes were enriched in several oncogenic signaling pathways, including TNFα signaling via NFkB, inflammatory response, interferon gamma response, KRAS signaling (UP), Complement, EMT, estrogen response, p53 pathway, IL6/JAK/STAT3 signaling, and coagulation (Figure S10B, Supporting Information).

Furthermore, hierarchical clustering analysis comparing ARID1A KO and ARID1B OE conditions revealed a clear cluster of genes that were similarly regulated upon ARID1A loss and ARID1B OE (Figure S10C, Supporting Information). Because ARID1B OE leads to reduced ARID1A levels, this finding suggests that ARID1B may regulate these genes indirectly through the downregulation of ARID1A. Such a mechanism could underlie ARID1B‐driven oncogenesis, wherein elevated ARID1B suppresses tumor‐suppressive ARID1A, thereby altering the ARID1A‐dependent transcriptome to promote tumorigenesis.

Moreover, GSEA analysis comparing ARID1B OE, ARID1B KO, and ARID1A KO further reveals both shared and distinct transcriptional changes that help elucidate ARID1B oncogenic mechanisms (Figure 4H, Figures S10D and S11, Supporting Information). Specifically, ARID1B OE resulted in significant upregulation of EMT and pro‐inflammatory pathways, including TNFα signaling via NFkB, interferon alpha response, interferon gamma response, and IL6/JAK/STAT3 signaling (Figure 4H, Figure S10D, Supporting Information). In contrast, ARID1A KO exhibited only modest or negligible changes in these pathways, suggesting that ARID1B exerts a stronger influence on EMT and pro‐inflammatory signaling than does ARID1A deficiency.

Glycolysis, hypoxia, and mTORC1 pathways were strongly downregulated in both ARID1A KO and ARID1B KO cells but showed only modest or no enrichment in ARID1B OE, suggesting that both ARID1A and ARID1B are required for the maintenance of these pathways and highlighting divergent metabolic impacts of ARID1A loss versus ARID1B OE (Figure S11A, Supporting Information). ARID1A KO exhibited significant upregulation of cell cycle‐related pathways, including the G2M checkpoint, E2F targets, and mitotic spindle, consistent with ARID1A tumor‐suppressive role. In contrast, ARID1B KO showed downregulation of these pathways, suggesting that ARID1B is required at normal levels for proper expression of genes involved in cell cycle regulation. ARID1B OE, meanwhile, showed modest upregulation of the mitotic spindle but also led to downregulation of the G2M checkpoint and E2F targets, possibly due to disruption of ARID1A‐containing complexes and a substantial shift in the transcriptional program (Figure S11A,B, Supporting Information).

To directly assess whether ARID1B localizes to regulatory regions of the oncogenic genes identified by RNA‐seq, we performed ChIP‐qPCR targeting the promoters of *TGFBI*, *ITGA5*, *TNC*, and *CXCL8*, which are enriched in EMT and inflammatory response pathways. Notably, these genes have been previously implicated in promoting tumor progression, invasion, and metastasis in various cancers, including breast cancer.^[^ ^42^, ^43^, ^44^, ^45^, ^46^, ^47^, ^48^, ^49^ ^]^ In ARID1B OE MDA‐MB‐468 cells, Flag‐ChIP revealed robust enrichment at all four promoters relative to both IgG control and Flag‐ChIP in control cells (Figure 4I), indicating that ectopically expressed ARID1B is directly recruited to these loci. To further validate endogenous ARID1B binding, we performed ChIP‐qPCR using an anti‐ARID1B antibody in ARID1B OE and ARID1B KO cells. We observed strong enrichment of ARID1B at *TNC* and *CXCL8* in ARID1B OE cells, which was significantly diminished in ARID1B KO (Figure 4J), confirming specific and ARID1B‐dependent chromatin occupancy. These findings provide direct evidence that ARID1B not only displaces ARID1A at selected loci but also independently occupies and transcriptionally activates key oncogenic targets.

Together, these results indicate that ARID1A and ARID1B regulate overlapping yet distinct transcriptional programs. Both factors are necessary to maintain hypoxia, glycolysis, and mTORC1 signaling. However, they diverge in their effects on cell cycle‐related genes. ARID1A restrains cell cycle progression, whereas ARID1B is required for proper expression of cell cycle genes. Importantly, our data suggest that ARID1B OE promotes tumorigenesis via two complementary mechanisms. It indirectly promotes tumorigenesis by downregulating the tumor‐suppressive ARID1A and its associated transcriptome, and it directly activates pro‐oncogenic pathways such as inflammatory signaling and EMT.

Additionally, to better understand the interplay between ARID1A and ARID1B, we performed ARID1A ChIP‐seq analysis in ARID1B KO and ARID1B OE cells (Figure S12, Supporting Information). As expected, ARID1A binding was significantly reduced in ARID1A KO cells, confirming the specificity of the identified peaks as bona fide ARID1A targets (Figure S12A, Supporting Information). Notably, ARID1A binding was lower in ARID1B OE cells compared to ARID1B KO, suggesting that ARID1B may antagonize ARID1A by competing for binding at specific genomic loci (Figure S12A, Supporting Information). For example, a prominent ARID1A peak upstream of the FGF1 transcription start site (TSS) was markedly reduced in ARID1B OE cells (Figure S12C, Supporting Information). A similar reduction in ARID1A occupancy was observed across the entire NT5E gene locus (Figure S12B, Supporting Information), further supporting ARID1B's ability to displace ARID1A. However, ARID1A binding at some loci appeared unaffected, as seen at the RUNX2 and CDKN1A genes (Figure S12D,E, Supporting Information), suggesting that ARID1B‐mediated antagonism is locus‐specific. Together, these findings indicate that ARID1B OE can reduce ARID1A chromatin binding at select target genes, potentially contributing to oncogenesis by suppressing ARID1A tumor‐suppressive functions.

### 2.5 Identification of Nuclear Transport‐Associated Proteins KPNA2, KPNB1, and RANBP2 as Critical Regulators of ARID1B Nuclear Import

**Figure 5:**
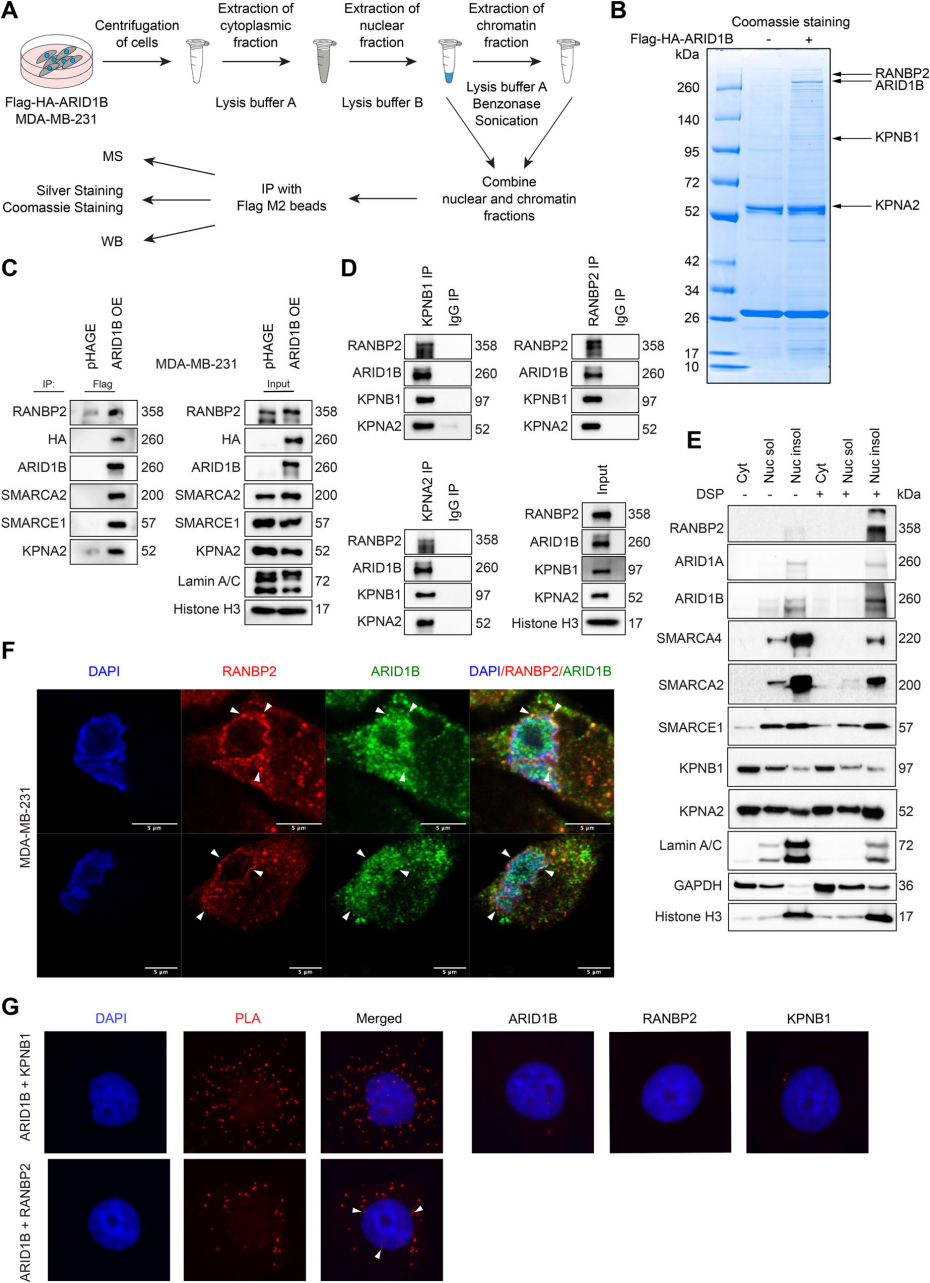
Identification of nuclear transport‐associated proteins KPNA2, KPNB1, and RANBP2 as critical regulators of ARID1B nuclear import. A) Schematic representation of the isolation and analysis of ARID1B complexes. MDA‐MB‐231 cells expressing Flag‐HA‐ARID1B were collected in a tube and centrifuged at low speed. The extraction of the cytosolic fraction was performed with lysis buffer A. The nuclear fraction was extracted with high salt lysis buffer B. The chromatin fraction was treated with benzonase and sonicated to shear the DNA. Protein complexes were pulled from the combined nuclear soluble and chromatin fractions using Flag M2 beads. The isolated protein complexes were subjected to Coomassie staining, mass spectrometry analysis, and immunoblotting. B) Coomassie staining of Flag M2 beads‐bound proteins. Flag M2 beads were incubated with nuclear extracts from empty vector control or Flag‐HA‐ARID1B MDA‐MB‐231 cells. Following incubation, the beads were washed, eluted with Flag peptide, boiled with SDS, and subjected to SDS‐PAGE gel electrophoresis for subsequent analysis using Coomassie staining. C) Nuclear extracts from empty vector control or Flag‐HA‐ARID1B MDA‐MB‐231 cells were employed for IP using Flag M2 beads. Subsequently, the eluted samples were separated by SDS‐PAGE gel electrophoresis and subjected to immunoblotting using the indicated antibodies. D) Nuclear extracts from MDA‐MB‐231 cells expressing Flag‐HA‐ARID1B were used for IP with the specified antibodies. Subsequently, the eluted samples were separated by SDS‐PAGE gel electrophoresis and subjected to immunoblotting using the indicated antibodies. E) Immunoblot analysis of the cytosolic (Cyt), nuclear soluble (Nuc sol), and nuclear insoluble (Nucl insol) fractions of MDA‐MB‐231 cells with or without dithiobis (succinimidyl propionate) (DSP) crosslinking. The purity of each fraction was assessed by detecting GAPDH, Lamin A/C, and Histone H3. F) ARID1B co‐localization with RANBP2 on the nuclear envelope. An immunofluorescence assay was conducted to detect the localization of ARID1B (*green*) and RANBP2 (*red*) in MDA‐MB‐231 cells. Nuclei were stained with DAPI (*blue*). The scale bars indicate 5 µm. G) Proximity ligation assays showing the interaction between ARID1B and RANBP2 and between ARID1B and KPNB1 in MDA‐MB‐468 cells.Identification of nuclear transport‐associated proteins KPNA2, KPNB1, and RANBP2 as critical regulators of ARID1B nuclear import. A) Schematic representation of the isolation and analysis of ARID1B complexes. MDA‐MB‐231 cells expressing Flag‐HA‐ARID1B were collected in a tube and centrifuged at low speed. The extraction of the cytosolic fraction was performed with lysis buffer A. The nuclear fraction was extracted with high salt lysis buffer B. The chromatin fraction was treated with benzonase and sonicated to shear the DNA. Protein complexes were pulled from the combined nuclear soluble and chromatin fractions using Flag M2 beads. The isolated protein complexes were subjected to Coomassie staining, mass spectrometry analysis, and immunoblotting. B) Coomassie staining of Flag M2 beads‐bound proteins. Flag M2 beads were incubated with nuclear extracts from empty vector control or Flag‐HA‐ARID1B MDA‐MB‐231 cells. Following incubation, the beads were washed, eluted with Flag peptide, boiled with SDS, and subjected to SDS‐PAGE gel electrophoresis for subsequent analysis using Coomassie staining. C) Nuclear extracts from empty vector control or Flag‐HA‐ARID1B MDA‐MB‐231 cells were employed for IP using Flag M2 beads. Subsequently, the eluted samples were separated by SDS‐PAGE gel electrophoresis and subjected to immunoblotting using the indicated antibodies. D) Nuclear extracts from MDA‐MB‐231 cells expressing Flag‐HA‐ARID1B were used for IP with the specified antibodies. Subsequently, the eluted samples were separated by SDS‐PAGE gel electrophoresis and subjected to immunoblotting using the indicated antibodies. E) Immunoblot analysis of the cytosolic (Cyt), nuclear soluble (Nuc sol), and nuclear insoluble (Nucl insol) fractions of MDA‐MB‐231 cells with or without dithiobis (succinimidyl propionate) (DSP) crosslinking. The purity of each fraction was assessed by detecting GAPDH, Lamin A/C, and Histone H3. F) ARID1B co‐localization with RANBP2 on the nuclear envelope. An immunofluorescence assay was conducted to detect the localization of ARID1B (*green*) and RANBP2 (*red*) in MDA‐MB‐231 cells. Nuclei were stained with DAPI (*blue*). The scale bars indicate 5 µm. G) Proximity ligation assays showing the interaction between ARID1B and RANBP2 and between ARID1B and KPNB1 in MDA‐MB‐468 cells.

To decipher the underlying mechanism by which ARID1B protein levels are regulated, we conducted M2 Flag beads purification of protein complexes coupled with mass spectrometry analyses to identify the ARID1B interactome (**Figure** **5**). We initially established stable expression of Flag/HA‐tagged hARID1B protein in MDA‐MB‐231 cells and conducted subcellular fractionation to purify ARID1B‐containing complexes from the nuclear fraction using empty vector cells as a control (Figures 2B and 5A). As illustrated in Figure 5B and Figure S13B (Supporting Information), we identified components of a cytonuclear transport system, including importins KPNA2 and KPNB1, as well as a subunit of the NPC RANBP2, as binding partners of ARID1B in breast cancer cells. A series of BAF complex subunit proteins were observed in the ARID1B interactome, including DPF2, BCL7C, SMARCA2, SMARCA4, ACTL6A, SS18, SMARCB1, SMARCC1, SMARCC2, SMARCD1, SMARCD2, SMARCD3, and SMARCE1 (Figure S13A, Supporting Information).^[^ ^13^, ^16^, ^18^, ^19^, ^20^, ^21^, ^22^, ^23^, ^33^, ^50^ ^]^ We further compared the ARID1B interactome in MDA‐MB‐231 cells with the Tang et al. dataset of proteins differentially expressed between breast cancer and adjacent non‐cancerous tissues.^[^ ^51^ ^]^ The result revealed that 145 out of 200 proteins (72.5%) found in the mass spectrometry data set from ARID1B pulldown are differentially expressed in breast cancer tissue compared to normal tissue, further supporting the oncogenic role of ARID1B in breast cancer (Figure S13C, Supporting Information).

To validate the above mass spectrometry results, we performed a series of co‐IP experiments, confirming the interaction between ARID1B, RANBP2, KPNA2, and KPNB1 in breast cancer cells (Figure 5C,D). Additionally, subcellular fractionation followed by immunoblot analysis showed that ARID1B, as well as other subunits of the BAF complex, are primarily enriched in the nuclear insoluble fraction, implying that these BAF subunits are predominantly associated with chromatin (Figure 5E). Importins KPNA2 and KPNB1 were found in all three fractions (cytosol, nuclear soluble, and nuclear insoluble) and were predominantly enriched in soluble fractions, which further supports their function as mobile nuclear transporters (Figure 5E). Immunostaining analysis demonstrated that ARID1B signals overlap with both KPNA2 and KPNB1 fluorescence signals (Figure S13D,E, Supporting Information). Moreover, RANBP2 was shown to co‐localize with ARID1B in the nuclear insoluble fraction (Figure 5E), with immunostaining followed by confocal microscopy showing significant overlap between ARID1B and RANBP2 signals at the nuclear envelope, suggesting that RANBP2 facilitates ARID1B cytosol‐to‐nucleus transport through the NPC (Figure 5F). To further confirm the interaction between ARID1B and its binding partners, we conducted a proximity ligation assay (PLA), which verified that ARID1B co‐localizes with KPNA2, KPNB1, and RANBP2 (Figure 5G, Figure S13F, Supporting Information).

### 2.6 Disruption of KPNA2–KPNB1–RANBP2‐Facilitated ARID1B Nuclear Translocation Alters Gene Expression and Suppresses Tumor Growth

**Figure 6:**
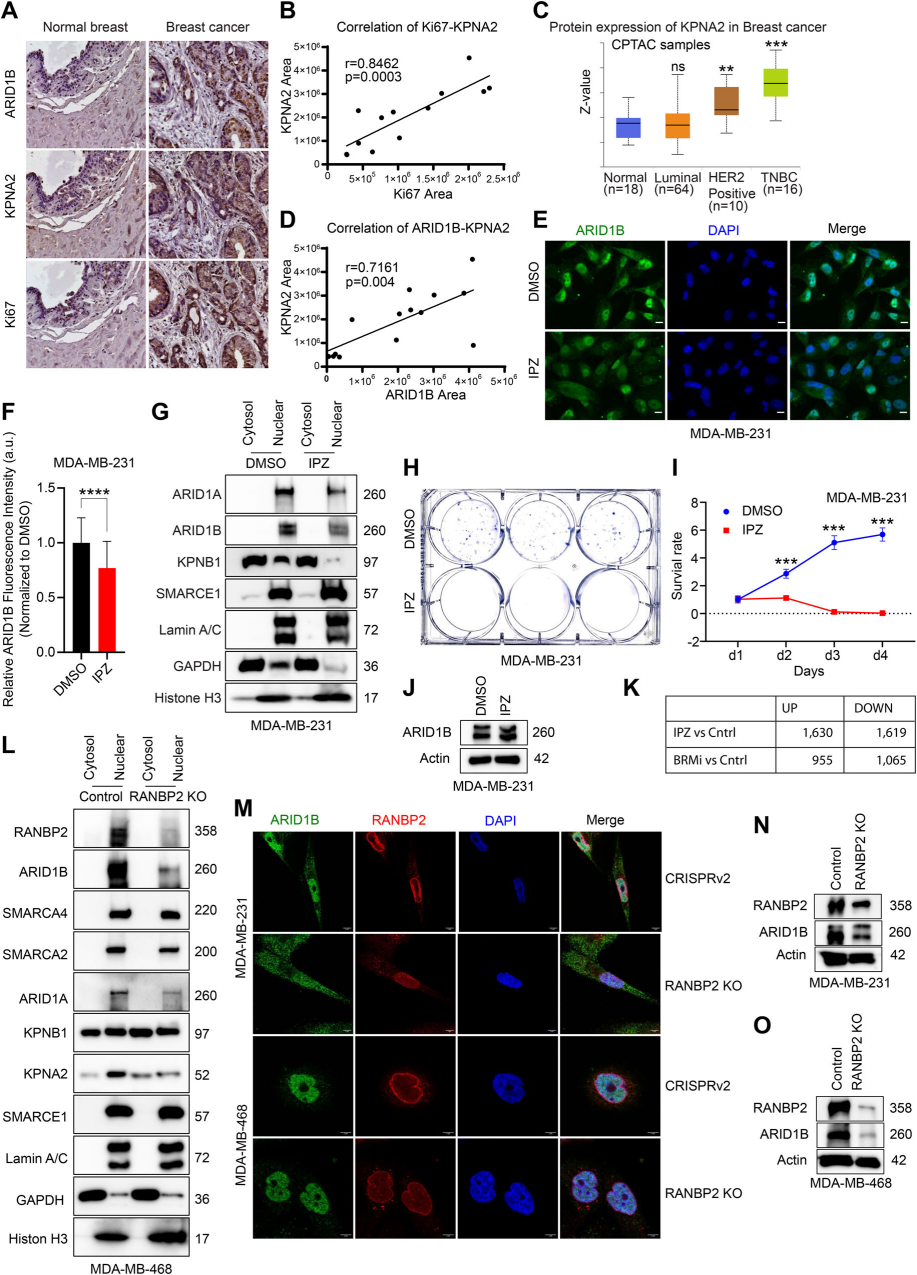
Disruption of KPNA2–KPNB1–RANBP2‐facilitated ARID1B nuclear translocation alters gene expression and suppresses tumor growth. A) 20X IHC of ARID1B, KPNA2, and Ki67 in breast cancer and normal tissues. B) Correlation of Ki67 and KPNA2 in breast cancer. C) Comparison of KPNA2 protein expression between normal tissue samples (*n* = 18) and tumor tissue samples from Luminal (*n* = 64), HER2+ (*n* = 10), and TNBC (*n* = 16) patients. Analysis of KPNA2 protein levels using CPTAC database and UALCAN data analysis portal showed significant accumulation of KPNA2 in HER2+ (*p* = 0.0097), and TNBC (*p* ≤ 0.0001), and no significant difference in Luminal (ns) compared to normal tissues.^[^ ^79^, ^80^ ^]^ *P*‐values are summarized with asterisks: *** – *p* ≤ 0.0001, ** – *p* ≤ 0.01, * – *p* ≤ 0.05. D) Correlation of ARID1B and KPNA2 in breast cancer. E) Immunofluorescence assay was conducted to detect levels of ARID1B (*green*) in MDA‐MB‐231 cells in response to IPZ treatment. Cells were treated with IPZ (40 µm) for 2 h. Nuclei were stained with DAPI (*blue*). The scale bars indicate 5 µm. F) Quantification of relative ARID1B fluorescence intensity in the nuclei of MDA‐MB‐231 cells after 2‐h IPZ treatment. G) Immunoblot analysis of cytosolic and nuclear fractions of MDA‐MB‐231 cells in response to IPZ treatment. MDA‐MB‐231 cells were treated with 40 µM IPZ or DMSO for 2 h. The protein levels of ARID1B, ARID1A, SMARCE1, and KPNB1 in the cytosolic and nuclear fractions were examined. Lamin A/C and Histone H3 were used as nuclear marker proteins. GAPDH was used as a cytoplasmic marker protein. Each lane was loaded with 30 µg of cytoplasmic or nuclear extract. H) Colony formation assay in response to continuous IPZ treatment in MDA‐MB‐231 cells. I) Survival rate of MDA‐MB‐231 cells under continuous IPZ treatment. Cell viability was determined using the CCK‐8 assay. The data is presented as the mean±SD of three independent experiments. Statistical significance was determined by two‐way ANOVA followed by the Bonferroni test. *P*‐values are summarized with asterisks: *** – *p* ≤ 0.0001 J) Immunoblot analysis of ARID1B following 2‐h IPZ (40 µM) treatment in MDA‐MB‐231 cells. Two‐hour IPZ treatment didnot affect total ARID1B levels. K) Differential gene expression in IPZ‐ or BRMi‐treated groups versus the control group. L) Immunoblot analysis of cytosolic and nuclear fractions of control and RANBP2 KO MDA‐MB‐468 cells. The protein levels of RANBP2, ARID1B, SMARCA4, SMARCA2, ARID1A, SMARCE1, KPNB1, and KPNA2 in the cytosolic and nuclear fractions were examined. Lamin A/C and Histone H3 were used as nuclear marker proteins. GAPDH was used as a cytoplasmic marker protein. Each lane was loaded with 30 µg of cytoplasmic or nuclear extract. M) Immunofluorescence assay was conducted to detect levels of ARID1B (*green*) and RANBP2 (*red*) in empty vector control (CRISPRv2) and RANBP2 KO breast cancer cell lines. Nuclei were stained with DAPI (*blue*). The scale bars indicate 5 µm. N,O) Immunoblot analysis of ARID1B in RANBP2 KO and empty vector control (Control) TNBC cells. RANBP2 KO resulted in a significant reduction of ARID1B in MDA‐MB‐231 N) and MDA‐MB‐468 O) cells.Disruption of KPNA2–KPNB1–RANBP2‐facilitated ARID1B nuclear translocation alters gene expression and suppresses tumor growth. A) 20X IHC of ARID1B, KPNA2, and Ki67 in breast cancer and normal tissues. B) Correlation of Ki67 and KPNA2 in breast cancer. C) Comparison of KPNA2 protein expression between normal tissue samples (*n* = 18) and tumor tissue samples from Luminal (*n* = 64), HER2+ (*n* = 10), and TNBC (*n* = 16) patients. Analysis of KPNA2 protein levels using CPTAC database and UALCAN data analysis portal showed significant accumulation of KPNA2 in HER2+ (*p* = 0.0097), and TNBC (*p* ≤ 0.0001), and no significant difference in Luminal (ns) compared to normal tissues.^[^ ^79^, ^80^ ^]^ *P*‐values are summarized with asterisks: *** – *p* ≤ 0.0001, ** – *p* ≤ 0.01, * – *p* ≤ 0.05. D) Correlation of ARID1B and KPNA2 in breast cancer. E) Immunofluorescence assay was conducted to detect levels of ARID1B (*green*) in MDA‐MB‐231 cells in response to IPZ treatment. Cells were treated with IPZ (40 µm) for 2 h. Nuclei were stained with DAPI (*blue*). The scale bars indicate 5 µm. F) Quantification of relative ARID1B fluorescence intensity in the nuclei of MDA‐MB‐231 cells after 2‐h IPZ treatment. G) Immunoblot analysis of cytosolic and nuclear fractions of MDA‐MB‐231 cells in response to IPZ treatment. MDA‐MB‐231 cells were treated with 40 µM IPZ or DMSO for 2 h. The protein levels of ARID1B, ARID1A, SMARCE1, and KPNB1 in the cytosolic and nuclear fractions were examined. Lamin A/C and Histone H3 were used as nuclear marker proteins. GAPDH was used as a cytoplasmic marker protein. Each lane was loaded with 30 µg of cytoplasmic or nuclear extract. H) Colony formation assay in response to continuous IPZ treatment in MDA‐MB‐231 cells. I) Survival rate of MDA‐MB‐231 cells under continuous IPZ treatment. Cell viability was determined using the CCK‐8 assay. The data is presented as the mean±SD of three independent experiments. Statistical significance was determined by two‐way ANOVA followed by the Bonferroni test. *P*‐values are summarized with asterisks: *** – *p* ≤ 0.0001 J) Immunoblot analysis of ARID1B following 2‐h IPZ (40 µM) treatment in MDA‐MB‐231 cells. Two‐hour IPZ treatment didnot affect total ARID1B levels. K) Differential gene expression in IPZ‐ or BRMi‐treated groups versus the control group. L) Immunoblot analysis of cytosolic and nuclear fractions of control and RANBP2 KO MDA‐MB‐468 cells. The protein levels of RANBP2, ARID1B, SMARCA4, SMARCA2, ARID1A, SMARCE1, KPNB1, and KPNA2 in the cytosolic and nuclear fractions were examined. Lamin A/C and Histone H3 were used as nuclear marker proteins. GAPDH was used as a cytoplasmic marker protein. Each lane was loaded with 30 µg of cytoplasmic or nuclear extract. M) Immunofluorescence assay was conducted to detect levels of ARID1B (*green*) and RANBP2 (*red*) in empty vector control (CRISPRv2) and RANBP2 KO breast cancer cell lines. Nuclei were stained with DAPI (*blue*). The scale bars indicate 5 µm. N,O) Immunoblot analysis of ARID1B in RANBP2 KO and empty vector control (Control) TNBC cells. RANBP2 KO resulted in a significant reduction of ARID1B in MDA‐MB‐231 N) and MDA‐MB‐468 O) cells.

Karyopherin alpha 2 (KPNA2), a member of the Karyopherin family of importins, facilitates the transportation of molecules essential for cell division, transcription, and DNA repair from the cytoplasm to the nucleus. KPNA2 recognizes the nuclear localization signal (NLS) on the cargo and recruits KPNB1 to transport it through the NPC.^[^ ^52^, ^53^, ^54^ ^]^ To better understand the mechanisms of ARID1B nuclear import, we studied the roles of importins and the NPC in regulating ARID1B. Our analysis showed that aberrant KPNA2 OE is directly correlated with aggressive breast cancer phenotypes, proliferative marker Ki67, and poor patient survival outcomes (**Figure** **6**A–C, Figure S14A, Supporting Information).^[^ ^55^, ^56^, ^57^ ^]^ A previous study showed that high expression of KPNA2 in breast cancer is also associated with reduced levels of DNA repair proteins, including CHK1, UBC9, PIAS1, BRCA1, RAD51, and γH2AX in cell nuclei and abnormally high levels of these proteins in the cytoplasm.^[^ ^56^ ^]^ Additionally, IHC staining conducted on a breast cancer tissue microarray revealed a positive correlation between the expression levels of ARID1B and KPNA2 (Figure 6A,D).

Karyopherin subunit beta 1 (KPNB1), also a member of the Karyopherin family of importins, plays an instrumental role in the nuclear import of various proteins that contribute to breast cancer progression, metastasis, and drug resistance.^[^ ^58^ ^]^ Recent studies also showed that KPNB1 is highly expressed in breast cancer and is associated with tumor progression and poor prognosis.^[^ ^57^, ^58^, ^59^ ^]^ To study the role of KPNB1 in regulating ARID1B, we examined the effect of KPNB1 blockade using the inhibitor importazole (IPZ). IPZ treatment for 2 h resulted in a significant reduction in nuclear ARID1B levels without affecting total ARID1B abundance and led to suppression of breast cancer cell growth. Prolonged treatment further enhanced this growth‐inhibitory effect (Figure 6E–J, Figure S14B,C, Supporting Information). Furthermore, it has been shown that tumor cell types exhibited heightened sensitivity to the knockdown of KPNB1 compared to various other importins, suggesting that elevated levels or activity of KPNB1 may play a crucial role in driving tumor progression.^[^ ^59^ ^]^ Due to high basal ARID1B expression and its long protein half‐life (∼48 h) in MDA‐MB‐468 cells, longer IPZ treatment was required to observe significant cytoplasmic enrichment and nuclear depletion of ARID1B by immunofluorescence (Figure S14D,E). To assess nuclear ARID1B levels by western blot, we performed 48‐h IPZ treatment with a lower dose (10 µM), which resulted in a significant reduction in ARID1B nuclear levels (Figure S14F, Supporting Information).

To delve deeper, we conducted RNA‐seq analysis following IPZ treatment in MDA‐MB‐468 cells. Our analysis revealed a total of 1630 and 1619 genes that were induced or repressed by IPZ, respectively (Fold change ≥ 2, p ≤ 0.05) (Figure 6K). Gene ontology (GO) analysis showed that IPZ‐induced genes were strongly enriched in TNFα, KRAS, and JAK/STAT3 signaling pathways, while IPZ‐repressed genes were involved in cell cycle, fatty acid metabolism, and xenobiotic metabolism (Figure S15A,B, Supporting Information).

As aforementioned, we discovered that RANBP2, a component of the NPC, interacts with ARID1B. To investigate its specific function in cytonuclear transport of ARID1B, we knocked out RANBP2 in TNBC cell lines using CRISPR technology (Figure 6L,M, Figure S16, Supporting Information). We discovered that depletion of RANBP2 resulted in a substantial decrease in the nuclear‐to‐cytosolic ratio of ARID1B (Figure 6L,M, Figure S16, Supporting Information). Importantly, we confirmed that prolonged IPZ treatment or RANBP2 KO also reduced total ARID1B protein levels, suggesting that impaired nuclear import leads to cytoplasmic degradation of ARID1B (Figure 6N,O). RANBP2 KO additionally reduced the nuclear levels of other large BAF complex components, including SMARCA2 (200 kDa) and ARID1A (260 kDa), while the nuclear levels of the smaller BAF subunit SMARCE1 (57 kDa) remained unchanged (Figure 6L, Figure S16E, Supporting Information). These findings highlight a potentially critical role for the NPC component RANBP2 in the nuclear translocation of larger BAF subunits.

To test the hypothesis that disruption of nuclear import results in cytoplasmic degradation of ARID1B, we treated MDA‐MB‐468 cells with the proteasome inhibitor MG132 and the lysosome inhibitor chloroquine (CQ). As shown in Figure S17A (Supporting Information), ARID1B levels increased following MG132 treatment, but not with CQ, indicating that ARID1B is primarily regulated by proteasomal degradation. Furthermore, treatment of RANBP2 KO and control MDA‐MB‐468 cells with MG132 led to robust accumulation of ARID1B in the cytoplasm, particularly in RANBP2 KO cells, further supporting that cytosolic ARID1B is unstable and subject to proteasomal degradation when its nuclear import is impaired (Figure S17B, Supporting Information).

To investigate whether nuclear import is essential for the oncogenic activity of ARID1B, we examined the functional consequence of disrupting nuclear import in ARID1B OE cells. Specifically, we pre‐treated ARID1B OE cells with IPZ, an inhibitor of KPNB1‐mediated nuclear import, for 2 h (Figure S18, Supporting Information). Colony formation assay revealed that IPZ pre‐treatment abolished the ARID1B‐induced increase in clonogenic growth in MDA‐MB‐231 cells (Figure S18A,B, Supporting Information). Cell viability assays also showed that IPZ treatment significantly decreased the growth rate of ARID1B OE cells (Figure S18C, Supporting Information). Consistently, IPZ treatment also abolished increased growth in ARID1B OE MDA‐MB‐468 cells (Figure S18D, Supporting Information), indicating that the oncogenic effects of ARID1B OE are dependent on the nuclear import machinery, particularly the KPNA2–KPNB1–RANBP2 complex.

In addition to RNA‐seq analysis following IPZ treatment in MDA‐MB‐468 cells, we also performed RNA‐seq analysis after KPNB1 KO to further assess its role in gene regulation (Figure S18E, Supporting Information). Differential expression analysis revealed that KPNB1 KO resulted in 504 upregulated and 699 downregulated genes in MDA‐MB‐468 cells (Figure S18F). Pathway enrichment analysis showed that these genes are involved in key cellular processes, including oxidative phosphorylation, Myc targets (v1), mitotic spindle, interferon alpha/gamma responses, glycolysis, mTORC1 signaling, G2/M checkpoint, hypoxia, and inflammatory response (Figure S18G, Supporting Information). Notably, these pathways strongly overlapped with those altered in ARID1B KO cells (Figure S9B, Supporting Information). Direct comparison between KPNB1 KO and ARID1B KO transcriptomes revealed 138 shared downregulated and 50 shared upregulated genes (Figure S18H, Supporting Information). These data further support the model that KPNB1‐dependent nuclear import is critical for ARID1B‐mediated transcriptional programs in breast cancer cells.

### 2.7 KPNB1 Pharmacological Inhibition Mimics the Effects of SWI/SNF Inhibitor on Chromatin Accessibility and Gene Expression

**Figure 7:**
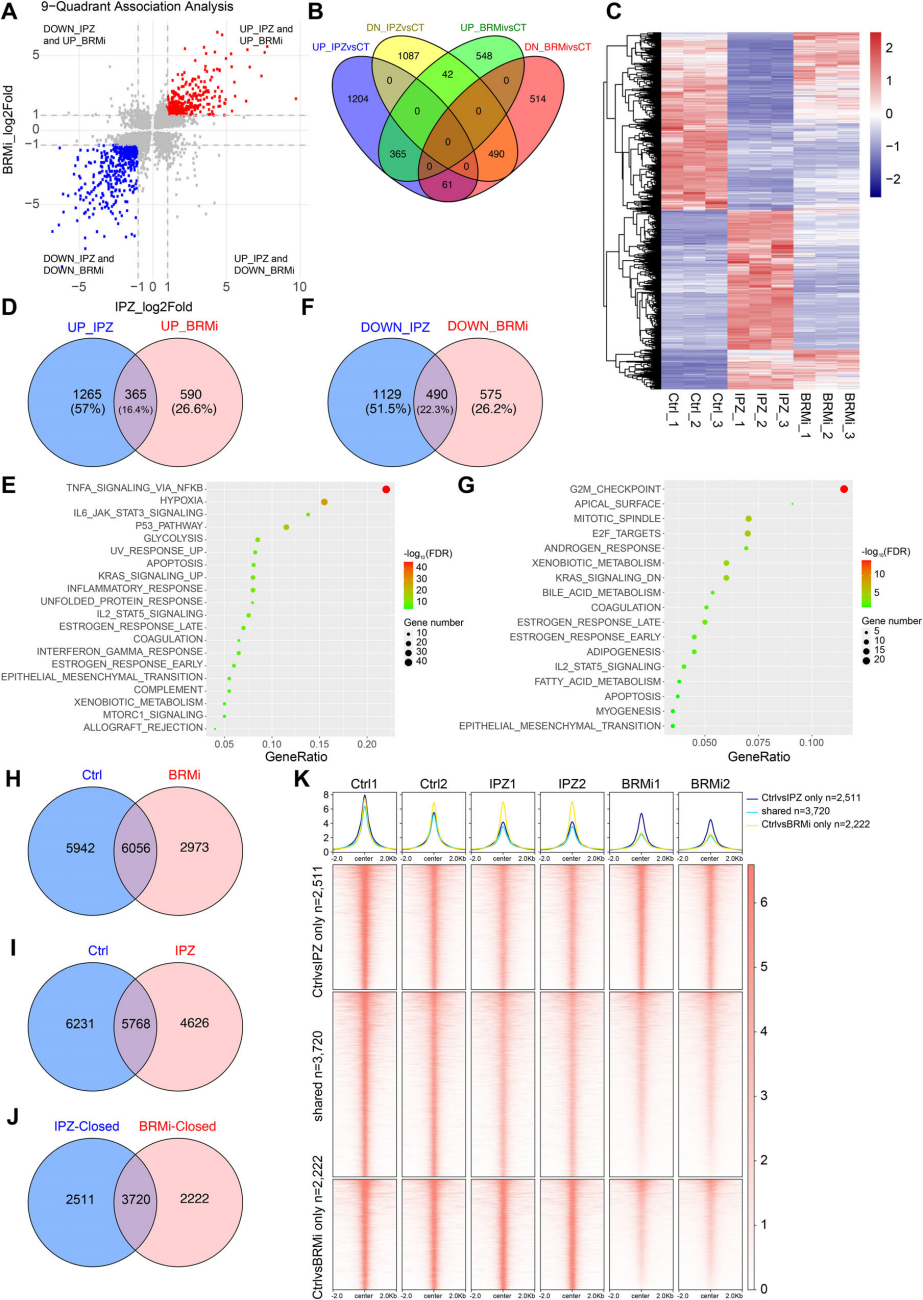
KPNB1 pharmacological inhibition mimics the effects of SWI/SNF inhibitor on chromatin accessibility and gene expression. A) Scatter plot demonstrates the correlation between changes in gene expression following treatment with KPNB1 inhibitor IPZ or with SWI/SNF inhibitor BRM014 (BRMi). Significantly altered genes (log2Fold ≥ 1 or log2Fold ≤ ‐1, adj. p ≤ 0.05) were distinguished by color, with upregulated genes marked in *red* (*n* = 365) and downregulated genes in *blue* (*n* = 490). The data were obtained from three biological replicates. B) Venn diagram represents overlapping upregulated and downregulated genes between BRMi‐treated and IPZ‐treated groups. C) Heatmap of upregulated (*red*) and downregulated (*blue*) genes after treatment with IPZ or BRMi in MDA‐MB‐468 cells. D,F) Venn diagram shows the overlap of upregulated D) and downregulated F) genes between IPZ‐ and BRMi‐treated cells. E,G) GO terms enrichment analysis with upregulated E) and downregulated G) genes in both IPZ‐ and BRMi‐treated cells. H,I) Venn diagram shows the overlap of ATAC peaks between control and BRMi‐treated groups H) and between control and IPZ‐treated groups I). J) Venn diagram shows the overlap of IPZ‐ and BRMi‐closed ATAC‐seq peaks. K) Matrix heatmap of ATAC‐seq peaks with reduced accessibility in response to IPZ and BRMi treatment.KPNB1 pharmacological inhibition mimics the effects of SWI/SNF inhibitor on chromatin accessibility and gene expression. A) Scatter plot demonstrates the correlation between changes in gene expression following treatment with KPNB1 inhibitor IPZ or with SWI/SNF inhibitor BRM014 (BRMi). Significantly altered genes (log2Fold ≥ 1 or log2Fold ≤ ‐1, adj. p ≤ 0.05) were distinguished by color, with upregulated genes marked in *red* (*n* = 365) and downregulated genes in *blue* (*n* = 490). The data were obtained from three biological replicates. B) Venn diagram represents overlapping upregulated and downregulated genes between BRMi‐treated and IPZ‐treated groups. C) Heatmap of upregulated (*red*) and downregulated (*blue*) genes after treatment with IPZ or BRMi in MDA‐MB‐468 cells. D,F) Venn diagram shows the overlap of upregulated D) and downregulated F) genes between IPZ‐ and BRMi‐treated cells. E,G) GO terms enrichment analysis with upregulated E) and downregulated G) genes in both IPZ‐ and BRMi‐treated cells. H,I) Venn diagram shows the overlap of ATAC peaks between control and BRMi‐treated groups H) and between control and IPZ‐treated groups I). J) Venn diagram shows the overlap of IPZ‐ and BRMi‐closed ATAC‐seq peaks. K) Matrix heatmap of ATAC‐seq peaks with reduced accessibility in response to IPZ and BRMi treatment.

Since ARID1B is a core subunit of SWI/SNF complex, it acts as a pivotal regulator of chromatin remodeling. To investigate whether KPNA2–KPNB1–RANBP2‐mediated ARID1B nuclear translocation affects chromatin accessibility and gene expression through the SWI/SNF complex, we conducted RNA‐seq analysis in MDA‐MB‐468 cells treated with BRM014 (BRMi). BRMi is a commonly used small‐molecule inhibitor that selectively targets the BRG1/BRM ATPase activity of the SWI/SNF complex.^[^ ^60^ ^]^ Interestingly, we observed that inhibition of BAF complex with BRMi resulted in a total of 955 upregulated and 1065 downregulated genes (Fold change ≥  2, *p* ≤ 0.05) (Figure 6K). GO analysis of these genes showed strong enrichment in similar signaling pathways and biological processes to those of KPNB1 inhibition in breast cancer (Figure S15A,B, S19A,B, Supporting Information). Further analysis showed a significant positive correlation between IPZ‐ and BRMi‐regulated genes (**Figure** **7**A), with a total of 365 upregulated genes and 490 downregulated genes (Figure 7B) by both treatments. Additionally, the heatmap also showed a similar co‐regulation by both treatments (Figure 7C). GO analyses of upregulated genes showed predominant enrichment in TNFα, JAK/STAT3, and p53 signaling pathways, as well as in hypoxia and glycolysis biological processes (Figure 7D,E), while downregulated genes were enriched in apical surface and cell cycle‐related biological processes (Figure 7F,G).

To further clarify the extent to which IPZ‐ and BRMi‐induced transcriptional changes are ARID1B‐dependent, we performed an in‐depth comparison with ARID1B expression‐dependent transcriptomes in breast cancer patients. Using ssGSEA analysis, we identified a strong inverse correlation between ARID1B‐high tumors and the gene expression programs repressed by IPZ and BRMi treatment. Specifically, pathways downregulated upon IPZ and BRMi treatment, including the mitotic G2M checkpoint, Ras signaling, and Notch signaling, were significantly upregulated in ARID1B‐high tumors (Figure S20, Supporting Information). Conversely, pathways upregulated by IPZ and BRMi, including apoptotic signaling, inflammatory response, and DNA damage response, were downregulated in ARID1B‐high tumors (Figure S20, Supporting Information). These results suggest that ARID1B contributes to oncogenic transcriptional programs in breast cancer, and that its inhibition by targeting nuclear import or SWI/SNF activity leads to a shift toward a tumor‐suppressive transcriptional state.

To assess whether IPZ and BRMi modulate similar chromatin regions, we performed ATAC‐seq analysis in MDA‐MB‐468 cells after treatment with either compound. Consistent with RNA‐seq results, we observed widespread decreases in chromatin accessibility: 5942 regions were less accessible upon BRMi treatment and 6231 regions upon IPZ treatment. Importantly, 3720 of these regions overlapped between the two treatments (Figure 7H–K), indicating a substantial convergence in chromatin remodeling outcomes. These data suggest that the changes in chromatin accessibility and gene expression observed following IPZ treatment are largely mediated through disruption of KPNA2–KPNB1–RANBP2‐mediated nuclear import of key SWI/SNF subunits, including ARID1B.

### 2.8 Computational Modeling of the Interactions Between ARID1B, Importin Subunits KPNA2 and KPNB1, and Nucleoporin RANBP2

**Figure 8:**
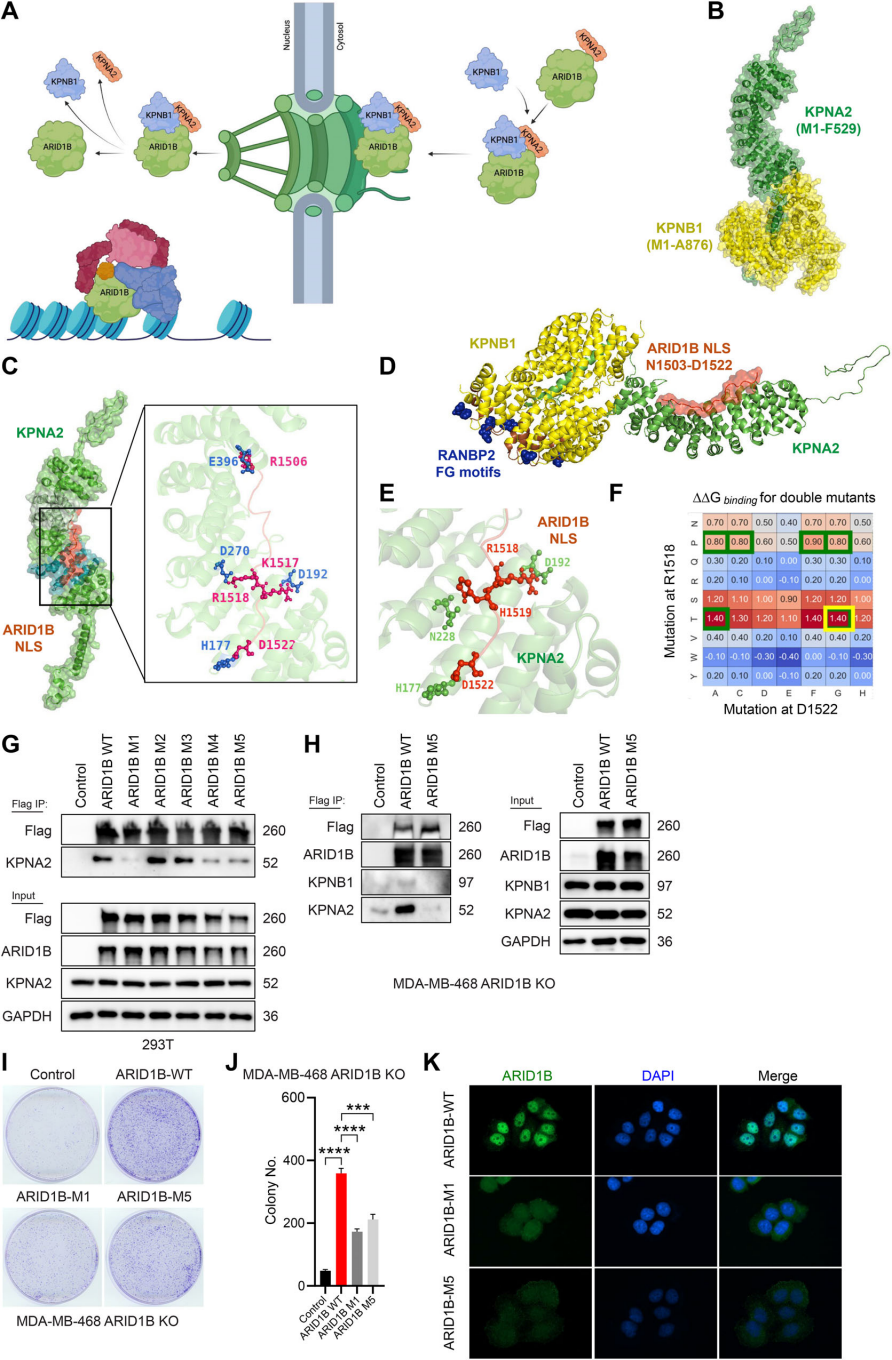
Computational modeling of the interactions between ARID1B, importin subunits KPNA2 and KPNB1, and nucleoporin RANBP2. A) Schematic representation of the protein–protein interactions involved in the nuclear translocation of ARID1B. ARID1B binds to the KPNA2–KPNB1 complex, which is then translocated to the nucleus. Created with BioRender.com. B) Structural model for the KPNA2 (*green*) and KPNB1 (*yellow*) heterodimer. C) Structural model for the complex formed between the NLS motif of ARID1B (*red*) and KPNA2. The NLS‐binding domain of KPNA2 consists of two subdomains, one major (W142‐R238 in *marine blue*) and the other minor (R315‐N403 in *light green*). The inset shows the ball and stick representation of ARID1B residues (*magenta*) forming salt bridges with KPNA2 residues (*marine blue*). D) Interaction of the FG motifs (*dark blue*) of RANBP2 with HEAT repeats 1–8 of KPNB1 complexed with KPNA2 and the ARID1B NLS. The interacting residues of KPNB1 are colored *brown*. E) ARID1B residues (*red* ball and stick) considered for mutagenesis studies. The corresponding KPNA2 residues involved in close interactions are shown in *green* ball and stick. F) A portion of the heatmap showing the change in free energy of ARID1B–KPNA2 binding due to amino acid substitutions at R1518 and D1522. The *green* boxes correspond to mutations in which the complex is destabilized by more than or equal to 0.8 kcal mol^−1^ and a ∆∆G_folding_ is less than or equal to 0.05 kcal mol^−1^. The selected pair for double mutation is shown in the *yellow* box. The complete heatmap is presented in Figure S23A, Supporting Information. G) Total lysates from HEK293T transfected cells were employed for IP using Flag M2 beads. Subsequently, the eluted samples were separated by SDS‐PAGE gel electrophoresis and subjected to immunoblotting using the indicated antibodies. H) Total lysates from ARID1B‐WT, ARID1B‐M5, and empty vector control (Control) MDA‐MB‐468 ARID1B KO cells were employed for IP using Flag M2 beads. Subsequently, the eluted samples were separated by SDS‐PAGE gel electrophoresis and subjected to immunoblotting using the indicated antibodies. I) Representative images of colony formation assay in ARID1B‐WT, ARID1B‐M1, ARID1B‐M5, and Control MDA‐MB‐468 ARID1B KO cells. J) Quantification of colony numbers formed in ARID1B‐WT, ARID1B‐M1, ARID1B‐M5, and Control MDA‐MB‐468 ARID1B KO cells. K) Immunofluorescence assay was conducted to detect levels of ARID1B (*green*) in ARID1B‐WT, ARID1B‐M1, and ARID1B‐M5 MDA‐MB‐468 ARID1B KO cells. Nuclei were stained with DAPI (*blue*). The scale bars indicate 30 µm.

To elucidate the nuclear import mechanism of ARID1B, we investigated its interactions with importin subunits KPNA2 and KPNB1, and NPC subunit RANBP2 in silico. **Figure** **8**A illustrates our proposed cytonuclear transport mechanism.

First, we modeled the KPNA2–KPNB1 heterodimer (Figure 8B). The importin‐β‐binding (IBB) domain of KPNA2 facilitates interfacial contacts during heterodimer assembly with KPNB1 (PDB: 1qgk; Figure S21A, Supporting Information).^[^ ^61^ ^]^ Although the complete structure of KPNA2 in complex with KPNB1 has not been fully resolved, we used AlphaFold2^[^ ^34^ ^]^ and RoseTTAFold^[^ ^62^ ^]^ to model KPNA2 and its complex with KPNB1. A RoseTTAFold‐predicted model closely matched the experimentally resolved IBB domain conformation complexed with KPNB1. The model was further refined using HADDOCK^[^ ^37^ ^]^ upon superimposing it onto the experimentally resolved IBB‐KPNB1 structure (Figure 8B).

Next, we modeled the RANBP2‐bound KPNB1 complex, taking into consideration the NLS motif of ARID1B that binds KPNA2 (Figure 8C,D). The NLS motif spans residues N1441‐D1460 (or N1503‐D1522 in the isoform considered). The KPNA2 epitope that binds this motif consists of a major domain (W142‐R238) and a minor domain (R315‐N403). The C‐terminal domain of ARID1B, containing the NLS motif, is disordered (segment P1413‐G1612) according to MobiDB‐lite^[^ ^63^ ^]^ and confirmed by AlphaFold2. Furthermore, the structures resolved for NLS motifs in complex with mouse KPNA2 (Figure S21B, Supporting Information) lack regular secondary structures. As a result, we focused on the NLS stretch (N1503‐D1522) to explore its binding mechanism with KPNA2, utilizing ClusPro^[^ ^64^ ^]^ and HADDOCK^[^ ^40^ ^]^ for guided docking simulations (Figure 8C). The resulting complex displayed an interaction surface area of 1306.1 Å^2^, a binding energy of −13.6 kcal mol^−1^, 24 hydrogen bonds (Figure S21C, Supporting Information), and four salt bridges (inset of Figure 8C,E) at the interface, aligning closely with the structures resolved for NLS motifs bound to mouse KPNA2.

The RANBP2 region spanning residues F1001‐G3206 contains 22 FG motifs that serve as recognition sites for binding the N‐terminal HEAT repeats 1–8 (residues 1–364) on the concave face of KPNB1. Given the intrinsic disorder in the RANBP2 C‐terminal domain, we aimed to identify a conformer that maximizes clustering of FG motifs facing the KPNB1 binding surface, promoting interfacial contacts. Iterative ClustENMD^[^ ^65^ ^]^ simulations for the segment P1401‐N2550 encompassing the FG motifs 4–15, using the AlphaFold2‐modeled RANBP2 structure, led to a conformation with five solvent‐exposed FG motifs within 30 Å of each other. The latter, used in guided docking simulations onto KPNB1 HEAT 1–8 repeats, yielded a complex model with a binding energy of −18.0 kcal mol^−1^ (computed by PRODIGY) (Figure S22A,B, Supporting Information). Figure 8D shows how KPNA2 and KPNB1 bridge the ARID1B and RANBP2 FG motifs.

To test the effect of interfacial interactions observed between ARID1B NLS motif and KPNA2, we focused on the residue pairs [R1518, D1522] and [R1518, H1519]. We scanned all possible mutations using SCWRL,^[^ ^66^ ^]^ generating heatmaps for changes in binding affinity (∆∆G_binding_) due to double mutations (Figure S23A,B, Supporting Information). Figure 8F illustrates the heatmap for the [R1518, D1522] interaction. The double mutant R1518T‐D1522G, as well as R1518T‐D1522A, resulted in a destabilizing ∆∆*G*_binding_ of 1.40 kcal mol^−1^, while maintaining NLS stability with a folding free energy change. In Figure S23B (Supporting Information), the heatmap for [R1518, H1519] demonstrates that the double mutant R1518T‐H1519G destabilizes the interface by ∆∆*G*_binding_ = 1.4 kcal mol^−1^ while increasing the NLS stability by 0.79 kcal mol^−1^. Furthermore, the triple mutant R1518T‐H1519G‐D1522G led to a ∆∆*G*_binding_ of 1.50 kcal mol^−1^ while maintaining NLS stability.

Figure 8G displays the IP results, which confirm that deletion of the N1503‐D1522 region disrupts the ARID1B–KPNA2 interaction. The double mutants M2 (R1518T‐H1519G) and M3 (R1518T‐D1522A) did not affect the interfacial interactions, whereas M4 (R1518T‐D1522G) and M5 (R1518T‐H1519G‐D1522G) significantly weakened them. These IP results underscore the critical role of R1518, H1519, and D1522 in mediating the ARID1B–KPNA2 binding. We further confirmed that the R1518T‐H1519G‐D1522G mutation disrupted the ARID1B–KPNA2–KPNB1 interaction in breast cancer cells (Figure 8H). To assess the functional implications, we conducted rescue experiments using a colony formation assay in breast cancer cells with ARID1B KO background.  ARID1B‐WT significantly increased the number of colonies, demonstrating a robust rescue effect (Figure 8I–K). In contrast, the rescue effect was significantly weaker when cells expressed either the NLS deletion mutant (M1) or the KPNA2‐binding deficient mutant (M5), although partial rescue was observed in these cases. These results indicate that the ARID1B–KPNA2 and nuclear localization are critical for the full functional activity of ARID1B in promoting colony formation.

### 2.9 Downregulation of ARID1B Reduces Tumor Growth and Augments the Therapeutic Efficacy of Niraparib in Eliminating Breast Tumors In Vivo

**Figure 9:**
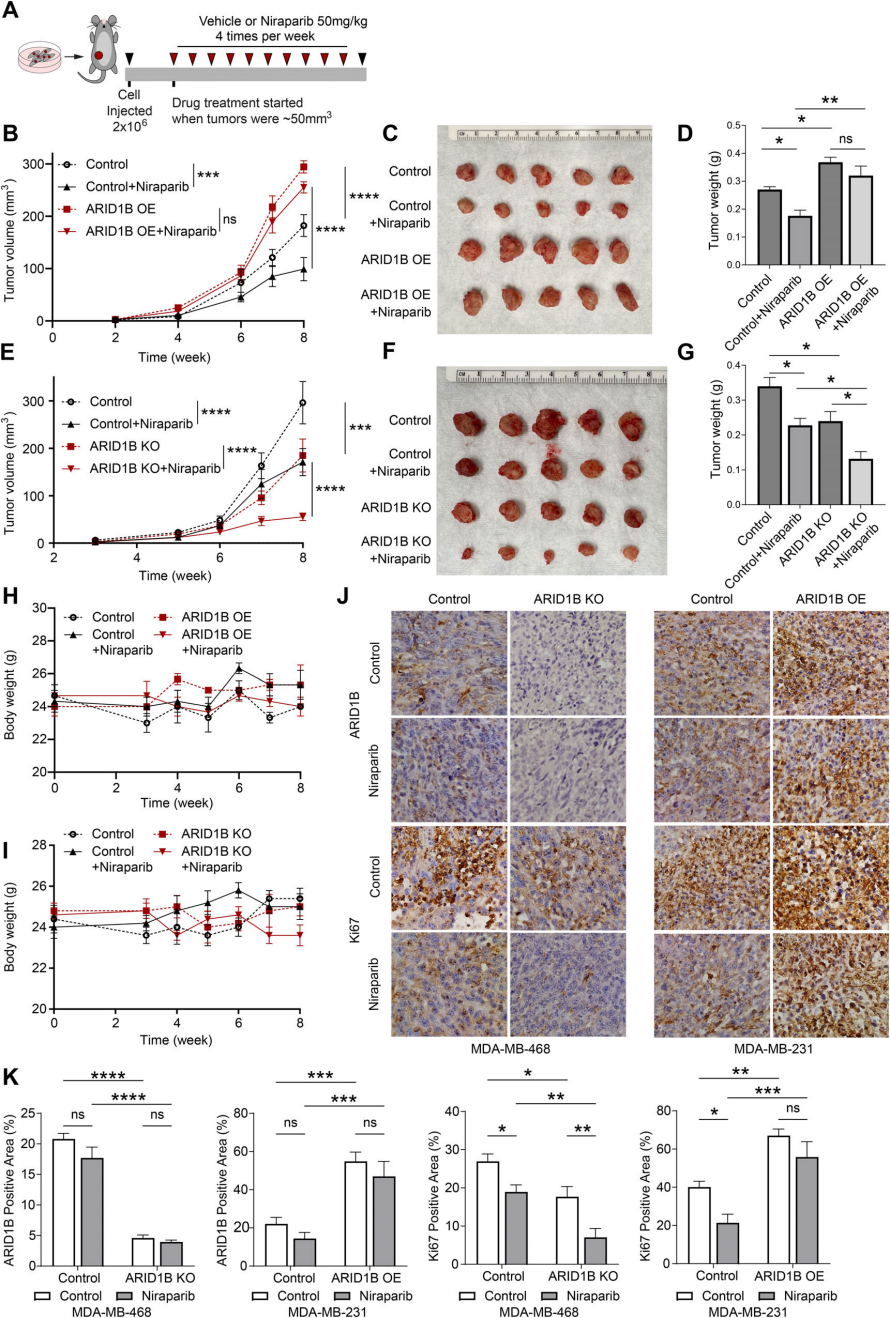
Downregulation of ARID1B reduces tumor growth and augments the therapeutic efficacy of Niraparib in eliminating breast tumors in vivo. A) Scheme of the animal experiment. B) Tumor growth of ARID1B OE and empty vector control (Control) MDA‐MB‐231 in female nu/nu mice measured for a period of 8 weeks. Tumor‐bearing mice were randomized into treated and non‐treated groups (*n* = 5) when the tumor reached a volume of 50 mm^3^. Mice were treated with Niraparib four times per week. One‐way ANOVA followed by the Tukey's multiple‐comparison test were performed for comparison of survival (ns, *P *> 0.05; *P < 0.05; **P < 0.01; ***P < 0.001; ****P < 0.0001). C,D) Size C) and weight D) of tumors formed in female nu/nu mice by ARID1B OE and Control MDA‐MB‐231 cells treated and non‐treated with Niraparib. E) Tumor growth of ARID1B KO and empty vector control (Control) MDA‐MB‐468 in female nu/nu mice measured for a period of 8 weeks. Tumor‐bearing mice were randomized into treated and non‐treated groups (*n* = 5) when the tumor reached a volume of 50 mm^3^. Mice were treated with Niraparib four times per week. One‐way ANOVA followed by the Tukey's multiple‐comparison test were performed for comparison of survival (ns, *P* > 0.05; *P < 0.05; **P < 0.01; ***P < 0.001; ****P < 0.0001). F,G) Size F) and weight G) of tumors formed in female nu/nu mice by ARID1B KO and Control MDA‐MB‐468 cells treated and non‐treated with Niraparib. H,I) Body weight of all four groups of mice from Figure 8B H) and Figure 8E I) was measured for a period of 8 weeks. J) 40X IHC of ARID1B and Ki67 in ARID1B KO and Control MDA‐MB‐468 tumors (left panel) and in ARID1B OE and Control MDA‐MB‐231 tumors (right panel) treated with Niraparib or Vehicle (Control). K) IHC analysis of ARID1B and Ki67 positive area (%) in ARID1B KO and Control MDA‐MB‐468 tumors and in ARID1B OE and Control MDA‐MB‐231 tumors treated with Niraparib or Vehicle (Control).Downregulation of ARID1B reduces tumor growth and augments the therapeutic efficacy of Niraparib in eliminating breast tumors in vivo. A) Scheme of the animal experiment. B) Tumor growth of ARID1B OE and empty vector control (Control) MDA‐MB‐231 in female nu/nu mice measured for a period of 8 weeks. Tumor‐bearing mice were randomized into treated and non‐treated groups (*n* = 5) when the tumor reached a volume of 50 mm^3^. Mice were treated with Niraparib four times per week. One‐way ANOVA followed by the Tukey's multiple‐comparison test were performed for comparison of survival (ns, *P *> 0.05; *P < 0.05; **P < 0.01; ***P < 0.001; ****P < 0.0001). C,D) Size C) and weight D) of tumors formed in female nu/nu mice by ARID1B OE and Control MDA‐MB‐231 cells treated and non‐treated with Niraparib. E) Tumor growth of ARID1B KO and empty vector control (Control) MDA‐MB‐468 in female nu/nu mice measured for a period of 8 weeks. Tumor‐bearing mice were randomized into treated and non‐treated groups (*n* = 5) when the tumor reached a volume of 50 mm^3^. Mice were treated with Niraparib four times per week. One‐way ANOVA followed by the Tukey's multiple‐comparison test were performed for comparison of survival (ns, *P* > 0.05; *P < 0.05; **P < 0.01; ***P < 0.001; ****P < 0.0001). F,G) Size F) and weight G) of tumors formed in female nu/nu mice by ARID1B KO and Control MDA‐MB‐468 cells treated and non‐treated with Niraparib. H,I) Body weight of all four groups of mice from Figure 8B H) and Figure 8E I) was measured for a period of 8 weeks. J) 40X IHC of ARID1B and Ki67 in ARID1B KO and Control MDA‐MB‐468 tumors (left panel) and in ARID1B OE and Control MDA‐MB‐231 tumors (right panel) treated with Niraparib or Vehicle (Control). K) IHC analysis of ARID1B and Ki67 positive area (%) in ARID1B KO and Control MDA‐MB‐468 tumors and in ARID1B OE and Control MDA‐MB‐231 tumors treated with Niraparib or Vehicle (Control).

Having developed a much clearer understanding of ARID1B function in vitro and the overall process of its nuclear translocation, we aimed to investigate ARID1B role in vivo. In particular, we were interested in its impact on breast cancer progression and drug response. To validate the role of ARID1B in tumorigenesis and drug response, we utilized preclinical mouse TNBC tumor models (**Figure** **9**A). We developed stable ARID1B OE and KO cell lines using MDA‐MB‐231 for OE and MDA‐MB‐486 cell line for KO, with an empty vector as a control. The impact on tumor growth was then assessed using NCr (nu/nu) athymic nude mice. Engineered empty vector control, ARID1B OE, and ARID1B KO cells were orthotopically injected into the mammary fat pads of female nu/nu mice (Figure 9A). We found that ARID1B OE significantly promoted tumor growth (Figure 9B–D). Treatment with the PARP inhibitor Niraparib significantly reduced tumor growth in the control group but not in the ARID1B OE group (Figure 9B–D). Additionally, ARID1B KO was shown to significantly suppress tumor growth, with an even more marked reduction in the ARID1B KO group treated with Niraparib (Figure 9E–G). Importantly, the use of Niraparib had no substantial effect on the weight of mice (Figure 9H,I). IHC analysis confirmed the expected expression patterns of ARID1B in the mouse tumors, with ARID1B KO tumors showing no detectable expression and ARID1B OE tumors exhibiting high levels of ARID1B (Figure 9J,K). Furthermore, ARID1B expression positively correlated with the proliferative marker Ki67, indicating the link between ARID1B levels and tumor cell proliferation.

Interestingly, in ARID1B KO tumors, Ki67 expression was almost absent in the Niraparib‐treated group compared to the vehicle‐treated group, suggesting a significant reduction in tumor cell proliferation in response to the drug. In contrast, ARID1B OE tumors maintained comparably high levels of Ki67 in both Niraparib‐ and vehicle‐treated groups, indicating that OE of ARID1B might confer resistance to the drug's anti‐proliferative effects (Figure 9J,K).

Finally, no changes were observed in the expression levels of KPNA2 or RANBP2 in response to either ARID1B KO or OE or to drug treatment, suggesting that these proteins are not directly influenced by ARID1B status or the drug in this context (Figure S24A, Supporting Information).

### 2.10 Disruption of ARID1B Nuclear Import Suppresses Breast Tumor Growth In Vivo

**Figure 10:**
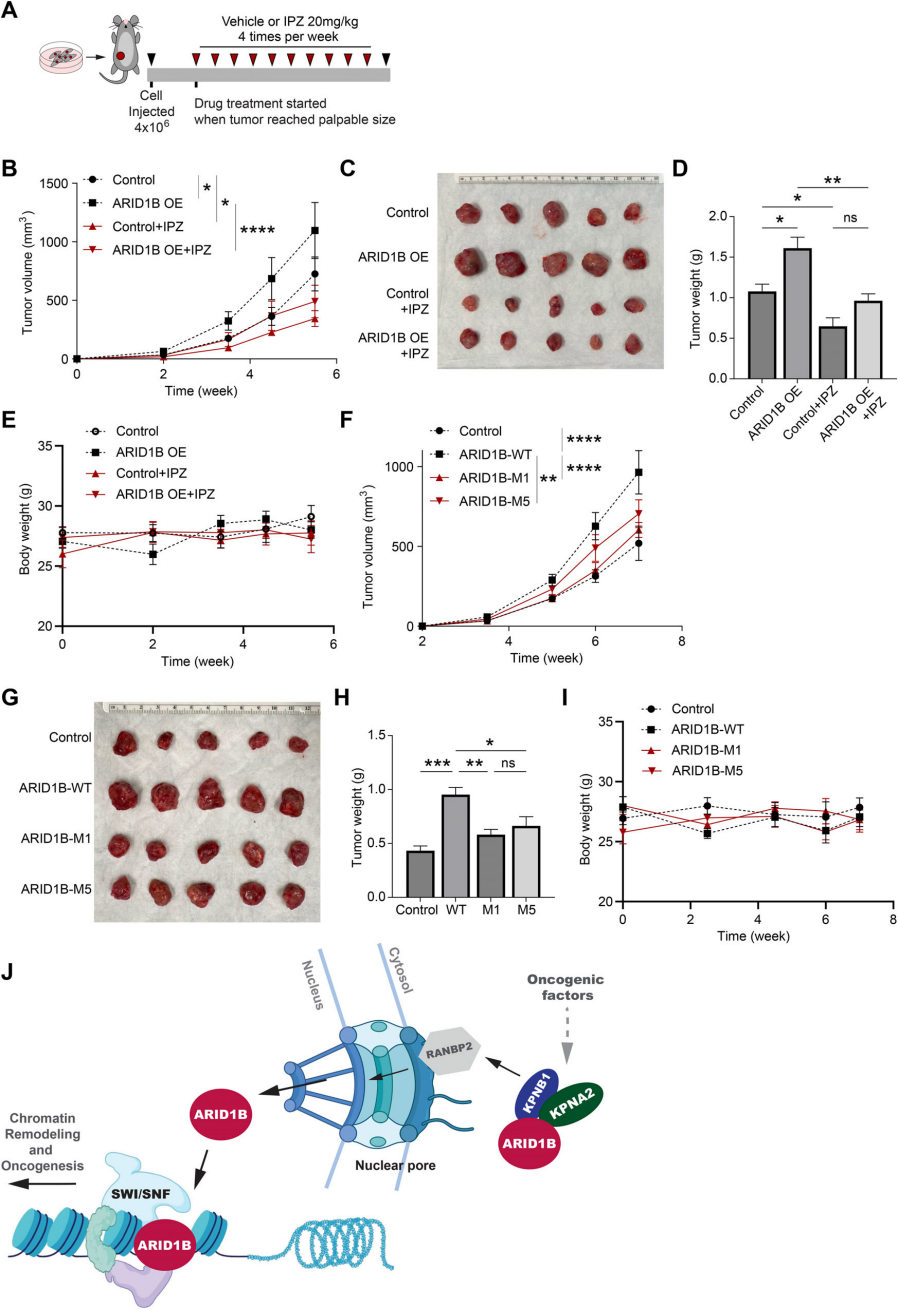
Disruption of ARID1B nuclear import suppresses breast tumor growth in vivo. A) Scheme of the animal experiment. B) Tumor growth of ARID1B OE and empty vector control (Control) MDA‐MB‐231 in female nu/nu mice measured for a period of 6 weeks. Tumor‐bearing mice were randomized into treated and non‐treated groups (n = 5) when the tumor reached a palpable size. Mice were treated with IPZ four times per week. One‐way ANOVA followed by the Tukey's multiple‐comparison test were performed for comparison of survival (ns, *P *> 0.05; *P < 0.05; **P < 0.01; ***P < 0.001; ****P < 0.0001). C,D) Size C) and weight D) of tumors formed in female nu/nu mice by ARID1B OE and Control MDA‐MB‐231 cells treated and non‐treated with IPZ. E) Body weights of all four groups of mice from B) tracked over a period of 6 weeks. F) Tumor growth of ARID1B‐WT, ARID1B‐M1, ARID1B‐M5, and empty vector control (Control) MDA‐MB‐468 ARID1B KO cells in female nu/nu mice measured for a period of 8 weeks. One‐way ANOVA followed by the Tukey's multiple‐comparison test were performed for comparison of survival (ns, *P* > 0.05; *P < 0.05; **P < 0.01; ***P < 0.001; ****P < 0.0001). G,H) Size G) and weight H) of tumors formed in female nu/nu mice by ARID1B‐WT, ARID1B‐M1, ARID1B‐M5, and Control MDA‐MB‐468 ARID1B KO cells. I) Body weights of all four groups of mice from F) tracked over a period of 8 weeks. J) Graphical summary of the ARID1B nuclear translocation mechanism, SWI/SNF complex formation, and its downstream chromatin remodeling function in breast cancer. Created with BioRender.com.Disruption of ARID1B nuclear import suppresses breast tumor growth in vivo. A) Scheme of the animal experiment. B) Tumor growth of ARID1B OE and empty vector control (Control) MDA‐MB‐231 in female nu/nu mice measured for a period of 6 weeks. Tumor‐bearing mice were randomized into treated and non‐treated groups (n = 5) when the tumor reached a palpable size. Mice were treated with IPZ four times per week. One‐way ANOVA followed by the Tukey's multiple‐comparison test were performed for comparison of survival (ns, *P *> 0.05; *P < 0.05; **P < 0.01; ***P < 0.001; ****P < 0.0001). C,D) Size C) and weight D) of tumors formed in female nu/nu mice by ARID1B OE and Control MDA‐MB‐231 cells treated and non‐treated with IPZ. E) Body weights of all four groups of mice from B) tracked over a period of 6 weeks. F) Tumor growth of ARID1B‐WT, ARID1B‐M1, ARID1B‐M5, and empty vector control (Control) MDA‐MB‐468 ARID1B KO cells in female nu/nu mice measured for a period of 8 weeks. One‐way ANOVA followed by the Tukey's multiple‐comparison test were performed for comparison of survival (ns, *P* > 0.05; *P < 0.05; **P < 0.01; ***P < 0.001; ****P < 0.0001). G,H) Size G) and weight H) of tumors formed in female nu/nu mice by ARID1B‐WT, ARID1B‐M1, ARID1B‐M5, and Control MDA‐MB‐468 ARID1B KO cells. I) Body weights of all four groups of mice from F) tracked over a period of 8 weeks. J) Graphical summary of the ARID1B nuclear translocation mechanism, SWI/SNF complex formation, and its downstream chromatin remodeling function in breast cancer. Created with BioRender.com.

Having demonstrated that ARID1B OE enhances tumor growth and confers resistance to PARP inhibition in vivo (Figure 9), we next investigated whether ARID1B nuclear localization is required for its tumor‐promoting function. To test this, we orthotopically injected control and ARID1B OE MDA‐MB‐468 cells into nude mice, followed by treatment with IPZ (**Figure** **10**A). Notably, IPZ treatment effectively abolished the tumor‐promoting effects of ARID1B OE, resulting in tumor growth comparable to the control group (Figure 10B–E).

To further determine whether the nuclear localization of ARID1B is essential for its tumor‐promoting function, we performed a rescue experiment using ARID1B KO MDA‐MB‐468 cells stably expressing either ARID1B‐WT or nuclear localization‐deficient mutants M1 and M5. Orthotopic injection into nude mice revealed that while ARID1B‐WT expression significantly restored tumor growth, neither M1 nor M5 mutant forms were able to rescue the ARID1B KO phenotype. Tumor growth in these mutant groups remained significantly suppressed and comparable to that of the ARID1B KO control group (Figure 10F–I). These findings highlight the critical role of ARID1B nuclear import, mediated via KPNA2 binding, in driving tumorigenesis in vivo.

## 3 Discussion

In this study, we elucidate the role of ARID1B in breast tumorigenesis and therapy by analyzing publicly available datasets from breast cancer patients, human specimens, conducting in vitro experiments, and utilizing animal models. Specifically, we demonstrated its tumor‐promotive role and its downstream effects on SWI/SNF complex functioning through the negative regulation of ARID1A expression and the disruption of ARID1A‐bound BAF complex formation. Additionally, we identified KPNA2–KPNB1–RANBP2 as a key protein cascade that regulates ARID1B nuclear import, highlighting its crucial role in chromatin remodeling and gene expression regulation (Figure 10J).

ARID1B is emerging as a potential therapeutic target because of its pivotal role in chromatin remodeling and its elevated levels in breast cancer, which typically indicate a worse prognosis.^[^ ^13^, ^16^, ^19^, ^21^, ^22^, ^23^, ^25^, ^26^, ^27^ ^]^ Thus, targeting ARID1B nuclear translocation could potentially hinder the survival and proliferation of cancer cells. Here, we demonstrated that depletion of ARID1B or inhibition of its nuclear translocation reduces tumor growth and enhances the therapeutic efficacy. While selective ARID1B inhibitors are still in early development, understanding the mechanism of ARID1B nuclear import and identifying specific amino acids responsible for its interaction with importing factors could help in the process of therapeutic development. Our analysis of the ARID1B interactome using protein complex purification coupled with mass spectrometry revealed regulators of nuclear translocation, including the importin subunits KPNA2 and KPNB1, as well as the NPC subunit RANBP2. Our findings collectively support a model of ARID1B cytonuclear transport mechanism in which KPNA2–KPNB1 heterodimer interacts with NLS of ARID1B, bringing it to RANBP2 of the NPC, and facilitating its nuclear import (Figure 10J). We confirmed that deletion of the NLS (N1503‐D1522) disrupts the interaction between ARID1B and KPNA2. Additionally, using computational modeling, we identified residues R1518, H1519, and D1522 to play a critical role in this interaction. Mutations in these residues [R1518T‐D1522G] and [R1518T‐H1519G‐D1522G] significantly weakened the interfacial interactions between ARID1B and KPNB1.

Inhibition of importin subunit KPNB1 by IPZ^[^ ^67^ ^]^ hindered ARID1B nuclear translocation, potentially disrupting SWI/SNF function in cancer cells. Here, we demonstrated that changes in chromatin accessibility and gene expression upon IPZ treatment significantly overlap with BRMi, which inhibits SWI/SNF activity. Moreover, we observed a significant reduction in TNBC cell viability and growth upon administration of IPZ in vitro. Although IPZ has not been tested in vivo for breast cancer, a study involving prostate cancer showed promising results when IPZ was injected intravenously into mice with prostate cancer cells. The treatment significantly reduced tumor size and weight, with minimal toxicity to normal cells expressing baseline KPNB1 levels.^[^ ^68^ ^]^ These findings suggest IPZ may be effective in treating breast cancer as well, though further optimization and preclinical studies are necessary to confirm its therapeutic potential and safety before clinical trials can be considered.

SWI/SNF complexes are composed of up to 15 subunits with interchangeable paralogues, resulting in a wide range of possible compositions that enable diverse functions depending on the cellular context.^[^ ^13^, ^16^, ^19^, ^20^, ^21^, ^22^, ^23^, ^69^ ^]^ Recent studies employing advanced imaging techniques such as cryogenic electron microscopy (cryo‐EM) have elucidated their three‐dimensional structure bound to the nucleosome. These studies confirm the established modular subunit organization and reveal interactions with the nucleosome.^[^ ^18^, ^33^, ^50^ ^]^ In this study, we demonstrated that the accumulation of ARID1B negatively impacts the formation of ARID1A‐bound BAF complexes, leading to a shift from tumor‐suppressive to oncogenic roles. This highlights a dynamic interplay within the SWI/SNF complex, where dysregulation of one component can affect its overall function and composition. However, the precise alterations in conformation and consequent effects on function need further investigation.

ARID1A and ARID1B are unique to canonical BAF (cBAF) complexes, each playing distinct roles as previously shown. In breast cancer, ARID1A is deficient while ARID1B is overexpressed.^[^ ^23^, ^25^, ^26^, ^28^ ^]^ Our study also revealed that ARID1B OE reduces ARID1A protein levels, suggesting contention for the BAF core between these two proteins. However, the mechanisms underlying this mutual exclusion are still not well‐defined. The low levels of ARID1A observed in breast cancer may be attributed to ARID1B higher affinity for the BAF core, increased ARID1A degradation, or both. This scenario suggests that ARID1A‐deficient cells may rely on ARID1B to maintain nuclear levels of BAF complex for their survival.^[^ ^13^, ^23^, ^27^ ^]^ However, further experimental validation, particularly in the context of breast cancer, is necessary to substantiate these findings.

In summary, developing inhibitors to specifically target ARID1B nuclear translocation shows promise for a more precise therapeutic approach in breast cancer, aiming to reduce off‐target effects linked with broader chromatin remodeling inhibitors. Careful consideration of specificity, efficacy, and clinical applicability is crucial for successful development and implementation. Combination therapies that target ARID1B nuclear import alongside PARP inhibitors could potentially enhance therapeutic outcomes.

Our findings suggest that ARID1B modulation may sensitize tumors to PARP inhibition, supporting a combinatorial strategy. Clinically, this approach may be particularly relevant for BRCA‐proficient TNBC subtypes that do not qualify for PARP inhibitor monotherapy. Furthermore, ARID1A‐mutant TNBCs, where ARID1B serves as a compensatory oncogenic driver, may represent a subset of patients most likely to benefit from dual ARID1B/PARP targeting. Biomarker‐guided selection based on ARID1A/ARID1B expression levels and mutational status will be essential for identifying responsive patient populations.

However, the high sequence similarity between ARID1B and ARID1A and the association of ARID1B loss with intellectual disability syndromes^[^ ^70^ ^]^ pose challenges for the development of small‐molecule inhibitors that are selectively ARID1B‐specific and can be precisely delivered to affected tissues.

Targeting ARID1B selectively within the BAF complex remains a significant challenge due to the high structural homology shared with ARID1A and the lack of enzymatic activity or well‐defined druggable pockets. One potential strategy to improve specificity includes structure‐based modeling to identify unique interaction interfaces or allosteric sites specific to ARID1B. In addition, the use of proteolysis‐targeting chimeras (PROTACs) or molecular glues that recruit E3 ligases selectively to ARID1B could offer an avenue for selective degradation.

Although our study establishes a compelling rationale for targeting ARID1B as a therapeutic vulnerability in ARID1A‐deficient breast cancers, further preclinical validation is warranted. Specifically, studies in patient‐derived xenograft (PDX) models and genetically engineered mouse models (GEMMs) are critical next steps to evaluate the in vivo efficacy and potential toxicity of ARID1B‐targeted therapies. Additionally, refining the specificity of ARID1B inhibitors, elucidating mechanisms of resistance, and identifying robust biomarkers of response will be essential for translating this strategy into a clinically actionable approach.

## 4 Experimental Section

### Cell Culture

184‐B5, MCF12A, MCF10A, MCF10DCIS.com, MDA‐MB‐468, HCC1937, SK‐BR‐3, AU‐565, HCC1954, BT474, MCF7, T47D, ZR‐75‐1, MDA‐MB‐436, MDA‐MB‐231, and HEK293T cells were obtained from the American Type Culture Collection (ATCC) (Manassa, VA). MDA‐MB‐468, HCC1937, SK‐BR‐3, BT474, MCF7, MDA‐MB‐436, MDA‐MB‐231, and HEK293T were maintained in Dulbecco's modified Eagle's medium (DMEM) supplemented with 10% fetal bovine serum (FBS), penicillin sodium (100 U mL^−1^), and streptomycin sulfate (100 µg mL^−1^). AU‐565, HCC1954, T47D, and ZR‐75‐1 cells were maintained in RPMI supplemented with 10% FBS, penicillin sodium (100 U mL^−1^), and streptomycin sulfate (100 µg mL^−1^). 184‐B5, MCF12A, MCF10A, and MCF10DCIS.com were maintained in DMEM/F12 supplemented with 5% horse serum, EGF (20 ng mL^−1^), hydrocortisone (0.5 µg mL^−1^), cholera toxin (100 ng mL^−1^), insulin (10 µg mL^−1^), penicillin sodium (100 U mL^−1^), and streptomycin sulfate (100 µg mL^−1^). All cell lines were cultured at 37 °C in a humidified atmosphere with 5% CO_2_.

### Antibodies and Reagents

The following antibodies were used: ARID1B (Abcam, ab‐57461), Ki67 (Cell Signaling Technology, 12202), actin (Sigma, A5441), GAPDH (Sigma, G8795), GAPDH (Novus Biologicals, NB300‐221), ARID1A (D2A8U) (Cell Signaling Technology, 123545), ARID1A (Santa Cruz Biotechnology, sc‐32761), SMARCE1 (Cell Signaling Technology, 33360), SMARCA2 (Cell Signaling Technology, 11966), SMARCA4 (Cell Signaling Technology, 49360), RANBP2 (Abcam, ab64276), RANBP2 (Santa Cruz Biotechnology, 74518), HA (C29F4) (Cell Signaling Technology, 3724), Lamin A/C (Cell Signaling Technology, 2032), Histon H3 (D1H2) (Cell Signaling Technology, 4499), KPNA2 (Santa Cruz Biotechnology, sc‐55538), KPNB1 (Abcam, ab2811), KPNB1 (ABclonal, A8610), Flag (Cell Signaling Technology, 14793S), Flag (Proteintech, 20543‐1‐AP).

Reagents used in this study are listed below: Niraparib (MK‐4827) (MedChemExpress, Cat. No. HY‐10619), Veliparib (ABT‐888) (MedChemExpress, Cat. No. HY‐10129), Tucatinib (TargetMol T2364), Neratinib (TargetMol T2325), SN38 (Cayman Chemical, 15632), benzonase nuclease (Sigma, E1014), Importazole (IPZ) (TargetMol 3445), BRM/BRG1 ATP Inhibitor‐1 (compound 14) (BRMi) (MedChemExpress, HY‐119374).

### Plasmids and Transfection

The plasmid pHAGE‐PGK‐N‐HA‐Flag‐ARID1B_NM_001374828.1‐Puro (pHAGE‐ARID1B) was custom‐generated by GenScript, USA Inc. to contain the full‐length of ARID1B with HA and Flag tags. Specifications for the custom sequence and subcloning were provided, and these tasks were executed by GenScript, USA Inc.

The ARID1B KO and RANBP2 KO cells were generated using pLenti‐CRISPRv2 (Adgene, plasmid 52961). Subcloning of ARID1B and RANBP2 sgRNAs into pLenti CRISPRv2 was performed by GenScript, USA Inc. Sequence of the sgRNAs are listed below: ARID1B sgRNA 1 GCGGCGCCTGCGTGTTCATC; ARID1B sgRNA 2 GGAAGCAACCAGTCTCGATC, ARID1B sgRNA 3 CTCTAGCCTGATGAACACGC; RANBP2 sgRNA 1 CTTTGCGTTCAAGTTTAGAA, RANBP2 sgRNA 2 AGCGATACACCTCCACTAGC, RANBP2 sgRNA 3 ACAAAGCCGTTGAATGTTAC.

For ARID1B mutagenesis, the corresponding mutations were introduced with primers via PCR amplification (2X Phanta Flash Master Mix P510 from Vazyme, ligated via Gibson assembly (ClonExpress MultiS One Step Cloning Kit C113 from Vazyme), and validated by restriction enzyme digestion and sequencing. Of note, the N‐terminal sequence of ARID1B was obtained by restriction digestion of the template with SalI and SpeI, rather than PCR, as this region could not be efficiently amplified, likely due to its high GC content.

### Lentiviral Infection

The expression plasmids pHAGE‐ARID1B and ARID1B KO were co‐transfected with pRRE, pRSV‐Rev, and pVSVg packaging plasmids into HEK293T cells using polyethyleneimine (PEI MAX, 24765, Polysciences Inc., Warrington, PA, USA). The medium containing packaged lentiviral particles was harvested 24 and 48 h after transfection and filtered through 0.45 µm filters. Human breast cancer cells were then infected with lentiviruses in the presence of polybrene (H9286, Sigma). To generate stable cell lines, virus‐infected cells were selected with 1 µg mL^−1^ of puromycin.

### Immunoblotting

Cells were washed with PBS and lysed using ice‐cold RIPA buffer supplemented with protease inhibitors (S8820, Sigma, Saint‐Louis, MO, USA). After centrifugation at 12000× *g* for 10 min at 4 °C, protein concentration was determined using the Pierce BCA Protein Assay Kit (Thermo Scientific, catalog No. 23225). Proteins were denatured by boiling in Laemmli sample buffer at 95 °C for 5 min, separated on 8% SDS‐PAGE gels, and transferred to 0.45 µm PVDF membranes. Membranes were blocked with PBST/5% milk for 1 h at room temperature. After overnight incubation at 4 °C with primary antibodies diluted in PBST/5% milk, membranes were incubated with anti‐mouse (Promega, W402B) or anti‐rabbit (Promega, W401B) HRP‐conjugated secondary antibodies diluted in PBST/5% milk for 1 h. Protein bands were developed using ECL detection reagents, visualized with the ChemiDoc system (Bio‐Rad Laboratories, Hercules, CA, USA), and analyzed using Image Lab software (Bio‐Rad Laboratories, Hercules, CA, USA).

### Cell Counting Kit‐8 (CCK‐8) Assay

Cells were seeded in 96‐well plates at a density of 5000 cells/well and incubated overnight at 37 °C. The cells were then further incubated for 24, 48, 72, and 96 h. Subsequently, 10 µL of the CCK‐8 reagent (TargetMol, C0005) was added to each well and incubated at 37 °C for 2 h. The absorbance of each well was measured at a wavelength of 450 nm using a microplate reader.

### Colony‐Formation Assay

Cells were seeded in 6 cm dishes at a density of 500–1000 cells per well. Once colonies (> 50 cells) were observed, the cells were fixed and stained with 0.1% crystal violet for 30 min at room temperature. Colony numbers were then analyzed.

### Transwell Migration Assay

Transwell assay inserts (pore size, 8 µm; Corning, Inc.) were used to evaluate the migration ability of control and ARID1B OE MDA‐MB‐231 cells. A total of 2.5 × 10^4^ cells in 0.2 mL of serum‐free medium were seeded into the upper chamber. Then, 750 µL of medium containing 10% FBS was added to the bottom chamber, and the plates were incubated for 24 h. Migrated cells were fixed with 70% ethanol and stained with 0.2% crystal violet for 20 min at room temperature. Images were taken at 4X magnification using the Lionheart FX automated microscope (BioTek), and the number of migrated cells was counted.

### RNA Extraction and qPCR

Total RNA (2 µg), extracted from breast cancer cells using the Trizol/Chloroform method, was used in the reverse transcription (RT) reaction to produce cDNA with the High‐Capacity cDNA RT Kit (Applied Biosystems Cat No 4374966). The RT reaction conditions were as follows: 10 min at 25 °C, 120 min at 37 °C, and 5 min at 85 °C. PCR was performed using the PowerTrack SYBR Green Master Mix (Applied Biosystems Cat No A46109) according to the manufacturer's instructions on the StepOne Plus real‐time PCR System (Applied Biosystems, Grand Island, NY). Relative expression to GAPDH was calculated using the 2^−∆∆Cq^ method. The specific primer sequences are listed as follows: Forward hARID1A ACCTCTATCGCCTCTATGTGTCTGT, Reverse hARID1A CTGGCAGCACTGCTTGATGT, Forward hGAPDH GCCAGCCGAGCCACAT, Reverse hGAPDH CTTTACCAGAGTTAAAAGCAGCCC.

### Immunoprecipitation (IP)

Cells were lysed in ice‐cold RIPA buffer supplemented with protease inhibitors (Sigma), and protein concentration was determined using the Pierce BCA Protein Assay Kit (ThermoFisher Scientific, 23225). For IP, cell lysates were incubated overnight at 4 °C with specific antibodies, followed by binding to Protein A (ThermoFisher Scientific, 10001D) or Protein G (ThermoFisher Scientific, 10003D) Dynabeads. Bound complexes were washed with PBS and eluted by boiling in Laemmli Sample Buffer (BioRad) at 95 °C for 10 min. Gel electrophoresis was conducted on 8% SDS‐PAGE gels, followed by transfer to PVDF membranes. Membranes were blocked with PBST/5% milk, incubated with primary antibodies overnight, and subsequently with either anti‐mouse (Promega, W402B) or anti‐rabbit (Promega, W401B) HRP‐conjugated secondary antibodies, or with VeriBlot for IP secondary antibody (Abcam, ab131366), or anti‐mouse IgG for IP (Abcam, ab13168).

### IHC

The human breast cancer tissue array was purchased from TissueArray.Com LLC. IHC staining of Ki67, ARID1B, and KPNA2 was performed using the IHC kit (Abcam) following the user manual. Briefly, slides were deparaffinized, rehydrated, and incubated in retrieval buffer (Sigma) for antigen retrieval, followed by staining procedures. Slides were blocked, incubated with primary antibodies, then incubated with the secondary HRP‐conjugated antibodies, and detected with the DAB detection kit (Abcam). IHC imaging was conducted using the ECHO Rebel microscope at 4X and 20X magnification. The images were analyzed using ImageJ software. Correlation analysis was performed using GraphPad Prism 10.0.

### Bioinformatics Analysis

Gene expression profiles were gathered from the TCGA‐BRCA study,^[^ ^71^ ^]^ which includes data from 1092 patients. The patients were classified into two groups based on ARID1B expression levels: ARID1B low group and ARID1B high group. The ARID1B low group comprised the top 5% of patients (55 patients) with the lowest ARID1B expression, while the ARID1B high group included the top 5% of patients (55 patients) with the highest ARID1B expression.

For ssGSEA,^[^ ^72^, ^73^ ^]^ pathways and gene sets were collected from Geno Ontology (GO)^[^ ^74^, ^75^ ^]^ annotations and the R package GSVA (v. 1.44.5)^[^ ^76^ ^]^ was employed to evaluate the enrichment score of pathways for each patient.

For Copy Number Variation (CNV) analysis, CNV data for breast cancer were accessed via the NCI Genomic Data Commons (GDC). Analyses were performed using the OncoMatrix web‐based tool (https://docs.gdc.cancer.gov/Data_Portal/Users_Guide/oncomatrix/).

For Proteomic Data Analysis, public TNBC datasets from CPTAC related to breast cancer were analyzed using established bioinformatics pipelines.^[^ ^77^, ^78^ ^]^ Protein IDs were clustered based on differences between the average expression in each subtype and other subtypes. Proteins related to the SWI/SNF complex, cell proliferation, and cell stemness/progenitor phenotype were specifically analyzed. Heatmaps were generated to visualize the expression levels, and Spearman's correlation coefficients were calculated to assess the significance of correlations among protein expression values in TNBC samples.

The online bioinformatics portal UALCAN (https://ualcan.path.uab.edu), which includes The Cancer Gene Atlas (TCGA) and the CPTAC databases, was utilized to analyze transcriptomic and proteomic data for breast cancer, respectively.^[^ ^79^, ^80^, ^81^ ^]^ Using this tool, the expression of ARID1B across various cancers, as well as in breast cancer major subtypes was analyzed, including Luminal, HER2+, and TNBC. A *p*‐value of less than 0.05 (*p* < 0.05) was considered to be statistically significant.

Kaplan–Meier survival curves were plotted using the Kaplan–Meier Plotter (Tang 2018 database) and the Xena platform (TCGA database), and statistical significance was analyzed using the log‐rank test.

### Computational Methods

Structures were modeled using available (partial) structures as templates and the tools AlphaFold2^[^ ^34^ ^]^ and RoseTTAFold,^[^ ^62^ ^]^ and refined using HADDOCK.^[^ ^37^ ^]^ Molecular docking simulations were performed using ClusPro^[^ ^64^ ^]^ and HADDOCK.^[^ ^40^ ^]^ Binding affinities (∆*G*_binding_) were evaluated using PRODIGY;^[^ ^82^ ^]^ the number of hydrogen bonds was computed using HBplus;^[^ ^83^ ^]^ and the number of salt bridges formed at the KPNA2‐ARID1B interface was computed using in‐house code. The interaction energy between ARID and the nucleosome in the BAF core was computed for both ARID1A and ARID1B subunits using FoldX.^[^ ^41^ ^]^ A total of 138 conformers were generated for the KPNA2‐ARID1B NLS complex, and 160 conformers for the KPNB1‐RANBP2 complex. Simulations of RANBP2 alternative conformers were conducted using ClustENMD^[^ ^65^ ^]^ with the AlphaFold2‐modeled structure of RANBP2.

For protein design scanning, ARID1B NLS residue pairs were chosen in such a way that i) both were present at the interface of a large fraction of ARID1B–KPNA2 complexes obtained by docking simulations and ii) they were located in each other's vicinity, which led to the pairs [R1518‐D1522] and [R1518‐ H1519] whose coupled substitutions were investigated. In order to determine the NLS double mutants that could potentially disrupt the formation of the complex ARID1B and KPNA2, all possible 19×19 substitutions were scanned at these particular NLS residue pairs using SCWRL.^[^ ^66^ ^]^ The interaction energies of the ARID1B–KPNA2 complexes (for the WT and mutant ARID1B) were computed using PRODIGY,^[^ ^82^ ^]^ and the change in the stability (∆G_folding_) of each ARID1B mutant relative to the WT protein was computed using FoldX.^[^ ^41^ ^]^ The objective was to find mutations that would give rise to weaker binding compared to that of the WT ARID1B–KPNA2 complex (−13.6 kcal mol^−1^) while minimally affecting the folding free energy of the ARID1B mutants.

### Subcellular Fractionation

For the preparation of cytosolic, nuclear, and chromatin fractions, cells were trypsinized and washed with PBS. After cells were spun down at 2000 rpm for 5 min, the cell pellet was lysed with buffer A (10 mm HEPES‐KOH, pH7.9, 10 mm KCl, 15 mm MgCl_2,_ 340 mm sucrose, 10% glycerol; supplemented with protease inhibitor, 10 mm sodium buturate, 0.2% Triton X‐100) on ice for 10 min and centrifuged at 1300 × *g* at 4 °C for 5 minu. After collection of the supernatant (cytosolic fraction) into an Eppendorf tube, the pellet was resuspended in buffer A with a high salt concentration (300 mM NaCl) and incubated on ice for 10 min, followed by a secondary centrifugation step at 1300 × *g* at 4 °C for 5 min. The supernatant (nuclear soluble fraction) was collected into a clean Eppendorf tube. The pellet (chromatin fraction) was resuspended in buffer A and subjected to sonication using a sonicator. All isolated fractions were then centrifuged at 20 000 × *g* at 4 °C for 20 min and used for further analysis. Note that before fractionation, cells were crosslinked with DSP (dithiobis (succinimidyl propionate)) (Thermo Fisher Scientific, PG82081), selectively applied in specific experiments.

### Purification of ARID1B Complexes and Mass Spectrometry

The combined nuclear soluble and chromatin fraction from MDA‐MB‐231 cells expressing Flag/HA‐ARID1B was obtained via subcellular fractionation. An IP assay using anti‐Flag M2 beads (Sigma, A2220) was performed to isolate ARID1B‐interacting proteins. The beads were washed four times with TBS buffer, and elution was initiated with 3X Flag peptide (Sigma, F4799) in TBS buffer. The eluates were either run on SDS‐PAGE gel followed by Coomassie staining or subjected to stacking gel electrophoresis and excised for mass spectrometry analysis.

Following analysis, the STRING online tool (https://string‐db.org) was employed to map identified proteins and create protein–protein interaction networks.

### Immunofluorescence (IF)

Immunofluorescence (IF) staining was conducted as described in the previous studies.^[^ ^84^ ^]^ Adherent cells were briefly washed in PBS, fixed in freshly made 4% paraformaldehyde, and permeabilized with 0.1% Triton X‐100. The cells were then quenched with 0.1 M glycine and blocked with PBST/1% (w/v) bovine serum albumin (BSA) for 1 h. Next, cells were incubated overnight at 4 °C with the appropriate primary antibody in a humidity chamber, followed by five washes with PBS (5 min each). Subsequently, cells were incubated with the appropriate fluorescein‐conjugated secondary antibody at room temperature in a dark, humidity chamber for 1 h. After incubating with DAPI (Thermo Fisher Scientific, EN62248) for 2 min, cells were mounted on glass slides by adding 20–30 µL of antifading agent/Vectashield (Vector Laboratories, H‐1700). Lionheart FX automated microscope (BioTek) was used for IF imaging. The fluorescein‐conjugated secondary antibodies used in this study are listed here: AF488 goat anti‐mouse IgG (Invitrogen, A11029), AF488 goat anti‐rabbit IgG (Invitrogen, A11034), AF594 goat anti‐rabbit IgG (Invitrogen, A32740), and AF594 goat anti‐mouse IgG (Invitrogen, A11032).

### Proximity Ligation Assay (PLA)

PLA was performed using the Duolink PLA kit (Sigma) following the manufacturer's instructions. Cells were seeded onto Ibidi 18‐well chamber slides (Ibidi, Germany). The next day, cells were fixed with freshly made 4% paraformaldehyde and permeabilized with 1% Triton X‐100. After fixation and permeabilization, cells were incubated with Duolink blocking solution for 1 h at 37 °C. Subsequently, primary antibodies were diluted in Duolink antibody diluent and incubated overnight at 4 °C. The following day, glass slides were washed three times with PBS at room temperature. PLA PLUS and MINUS probes were diluted in Duolink antibody diluent and applied to the slides, incubating for 1 h at 37 °C. Slides were washed again three times with PBS at room temperature. After washing, Duolink ligation buffer was diluted in high‐purity water, ligase was added, and the ligation solution was applied to the slides for 30 min at 37 °C. After another round of washing, amplification buffer was diluted in high‐purity water, polymerase was added, and amplification was performed for 100 min at 37 °C. Following three subsequent washes at room temperature, cells were mounted with Duolink in situ mounting medium with DAPI before visualization with a Lionheart FX automated microscope (BioTek).

### 3D Mammosphere Formation Assay

Cells were seeded at a density of 1 × 10^3^ cells per well in 96‐well ultra‐low attachment plates (Corning) using 3D mammosphere culture medium (Mammocult, Millipore–Sigma, Cat# C‐28070) to prevent adherence. Plates were incubated in a humidified incubator at 37 °C with 5% CO₂ for 5–10 days. Mammospheres were visualized and counted using a Leica Thunder Widefield Microscope.

### Cancer Stem Cell Marker Analysis by Flow Cytometry

To evaluate cancer stem cell self‐renewal capacity, mammospheres formed by ARID1B OE and control MDA‐MB‐231 and MDA‐MB‐468 cells were collected and dissociated into single‐cell suspensions using 0.05% trypsin‐EDTA. Cells were washed and stained with PE‐conjugated anti‐human CD44 (BD Biosciences, Cat# 555479), FITC‐conjugated anti‐human CD24 (BD Biosciences, Cat# 555427), and Zombie Yellow live/dead viability dye (BioLegend) following the manufacturer's protocols. Samples were acquired on a Cytek Aurora flow cytometer, and data were analyzed using FlowJo software (BD Biosciences). The percentage of CD44⁺/CD24⁻ cells among the live cell population was quantified to assess stem‐like properties.

### ALDH1 Assay

Aldehyde dehydrogenase 1 (ALDH1) activity was measured using the ALDEFLUOR Kit (StemCell Technologies, Cat# 01700) according to the manufacturer's instructions. Briefly, mammospheres were trypsinized into single‐cell suspensions and incubated with ALDEFLUOR reagent in the presence or absence of the ALDH inhibitor diethylaminobenzaldehyde (DEAB) as a negative control. Zombie Yellow viability dye was included to exclude dead cells. Samples were analyzed on a Cytek Aurora flow cytometer, and the percentage of ALDH1⁺ cells among the live population was calculated using FlowJo software.

### RNA‐Seq Data Analysis

Total RNA was extracted from cells using the RNeasy Plus Mini kit following the manufacturer's instructions (Qiagen Inc., Valencia, CA). RNA‐seq reads were mapped to the NCBI human genome GRCh38 using STAR. Raw counts of genes were calculated by STAR. FPKM (Fragments Per Kilobase of transcript per Million mapped reads) values were calculated using the in‐house Perl script. Differential gene expressions were analyzed with the R Bioconductor DESeq2 package, which uses shrinkage estimation for dispersions and fold‐changes to improve the stability and interpretability of estimates.

Heatmaps were generated using the R package pheatmap. GO and GSEA pathway analyses were performed using MSigDB and visualized with the R/Bioconductor packages clusterProfiler, enrichplot, and ggplot2.

### ATAC‐Seq Data Analysis

The assay for transposase‐accessible chromatin using sequencing (ATAC–seq) was performed as previously reported.^[^ ^85^ ^]^ Briefly, 50 000 cells were washed once with 1X PBS and resuspended in 100 µl of lysis buffer (10 mM Tris‐HCl, pH 7.4, 10 mm NaCl, 3 mM MgCl_2_, 0.1% NP 40, 0.1% Tween‐20, 0.01% Digitonin) for 3 min on ice, followed by adding 1 ml of wash buffer (10 mM Tris‐HCl, pH 7.4, 10 mM NaCl, 3 mM MgCl_2_, 0.1% Tween‐20) to remove the cytoplasm and mitochondria fraction. The nuclei pellet was resuspended in 25 µl transposition mix (12.5 µl of 2X TD buffer, 2.5 µl of Tn5 Transposome, 8.25 µL of 1X PBS, 0.25 µL of 1% digitonin, 0.25 µL of 10% Tween‐20, 1.25 µl H_2_O) and incubated at 37 °C for 30 min in a thermomixer with 300 rpm. The fragmented DNA was purified using the DNA Clean & Concentrator‐5 kit (ZYMO Research). Libraries were amplified, and adapter and primer dimers were removed. Paired‐end sequencing was performed using an Illumina NovaSeq X Plus.

FastQC was used to check data quality. ATAC‐seq reads were aligned to the Human Reference Genome (assembly hg38) using Bowtie2. PCR duplicate reads were removed using Picard. Adaptors were trimmed with Trimmomatic. ATAC‐seq peak identification, overlapping, subtraction, and feature annotation of enriched regions were performed using the HOMER (Hypergeometric Optimization of Motif EnRichment) suite. Weighted Venn diagrams were created using the R package Vennerable. Peak profiles and heatmaps were generated by deepTools. Publicly available ChIP‐seq datasets were analyzed by Bowtie2 and MACS2.

All sequencing data (RNA‐seq and ATAC‐seq) generated for the study are deposited in the Gene Expression Omnibus at https://www.ncbi.nlm.nih.gov/geo/query/acc.cgi.

### Chromatin Immunoprecipitation (ChIP), ChIP‐Seq, ChIP‐qPCR

ChIP and ChIP‐seq were performed as previously described.^[^ ^86^, ^87^, ^88^, ^89^ ^]^ The following antibodies were used: anti‐ARID1A (CST 12354), anti‐ARID1B (Abcam 57461), and anti‐Flag (CST 14793). The specific primer sequences are listed as follows: Forward hCXCL8 ATGATTGGCTGGCTTATCTTCAC, Reverse hCXCL8 GAGTGGCAGGTGTTAGAACAAGA, Forward hTNC TGGGTTATCTCTCCGCTGGC, Reverse hTNC CACCTCGGAGCGAGTACAGG, Forward hITGA5 CCTCATTAGGAAATTCTCCGCTC, Reverse hITGA5 GTTAGACTGGGCGGGTTTGG, Forward hTGFBI GCCCAGCTTCCCCGC, and Reverse hTGFBI CGAGCGAGCTAGCGACC.

### ChIP–Seq Data Analysis

Sequencing reads were aligned to the Human Reference Genome (assembly hg38) using Bowtie2. ChIP–seq peaks were identified by MACS2 and feature annotation of enriched regions was performed using the HOMER suite. Heatmap views of ChIP–seq were generated by deepTools.

### Animal Experiments

All animal studies were conducted in accordance with the guidelines and approval of Emory University Institutional Animal Care and Use Committee (approved IACUC protocol number PROTO202200164). Female 6‐week‐old NCr (nu/nu) athymic nude mice were purchased from The Jackson Laboratory. ARID1B OE MDA‐MB‐231, ARID1B KO MDA‐MB‐468 cells, and their corresponding empty vector controls (2 × 10^6^ cells) were orthotopically injected into the mammary fat pad. Tumor volumes were calculated according to the equation: (length) x (width)^2^/2. Once tumors reached palpable size (approximately 50 mm^3^), mice were orally administered 50 mg/kg of Niraparib (Selleck Chemicals LLC, S2741) or vehicle control four times per week. Tumor size and body weight were measured twice per week. Eight weeks after tumor injection, the mice were sacrificed, and tumors were excised and weighed.

For IPZ‐treatment and ARID1B‐mutants animal experiments, ARID1B OE MDA‐MB‐231, ARID1B‐Mutant M1, and M5 MDA‐MB‐468 cells, and their corresponding empty vector controls (4 × 10^6^ cells) were orthotopically injected into the mammary fat pad. Tumor volumes were calculated according to the equation: (length) x (width)^2^/2. Once tumors reached palpable size, mice were administered 20 mg/kg of IPZ or vehicle control four times per week.

### Statistical Analysis

All quantitative data points represent the mean of three independent experiments performed in duplicates or triplicates with standard deviation (S.D.). Unless otherwise indicated, statistical analysis was performed using the two‐tailed Student's *t*‐test in GraphPad InStat (Graphpad Software, Inc., La Jolla, CA). *p < 0.05, **p < 0.01, and ***p < 0.001 were considered to indicate statistically significant differences and *p* > 0.05 was considered not significant (ns).

## Conflict of Interest

The authors declare no conflict of interest.

## Author Contributions

O.O. and Y.W. conceived the project. I.B. and Y.W. supervised research. O.O., X.C., L.Z., C.L., and X.L. performed the experiments and analyzed the results. O.O., A.B., Y.Z., M.Z., and J.C.Z. performed bioinformatics and computational analyses. O.O., A.B., X.C., C.L., Y.Z., M.Z., and J.C.Z. produced figures with input from X.L., J.Y., I.B., and Y.W. X.L., L.W., J.Y., and I.B. guided with methodology and oversaw bioinformatics and computational analyses. O.O., A.B., C.L., I.B., and Y.W. wrote the manuscript with input from all authors. O.O., A.B., X.C., L.Z., A.U., C.L., Y.Z., M.Z., X.L., N.S.Y., J.Y., J.C.Z., and Y.W. contributed to the revision of the manuscript. All authors discussed the results and commented on the manuscript.

## Supporting information



Supporting Information

## Data Availability

All data needed to evaluate the conclusions in the paper are present in the paper and/or the Supplementary Materials.
