# Supplementary material for: Disruption of ARID1B Recruitment to the Nuclear Pore Complex as a New Anticancer Therapeutic Strategy
Source: Adv Sci (Weinh). 2025 Jul 16;12(36):e15585. doi: 10.1002/advs.202415585 (PMC12463049; doi:10.1002/advs.202415585)
Supplement: Supplementary file 1 — Supporting Information [file ADVS-12-e15585-s001.docx]

Supporting Information

Disruption of ARID1B recruitment to the nuclear pore complex as a new anticancer therapeutic strategy

Olena Odnokoz, Anupam Banerjee, Xin Cui, Lidan Zeng, Amad Uddin, Christopher Li, Yueming Zhu, Mengyuan Zhang, Xiaodong Lu, Nagendra S. Yarla, Lu Wang, Jindan Yu, Jonathan C. Zhao, Ivet Bahar, Yong Wan*

Figure S1. Elevated expression of ARID1B correlates with a poor prognosis of breast cancer. (A) Comparison of ARID1B protein expression in tumor vs. normal samples across various cancers was conducted using the Clinical Proteomic Tumor Analysis Consortium (CPTAC) database and UALCAN data analysis portal.^[79–81]^ *Turquoise* bars represent normal tissue samples, while *pinkish-red* bars represent cancer tissue samples. The analysis showed an increased ARID1B expression in breast cancer (p ≤ 0.0001) and glioblastoma (p ≤ 0.0001) but not in other types of cancer. P-values are summarized with asterisks: *** - p ≤ 0.0001, ** - p ≤ 0.001, * - p ≤ 0.05. (B) Comparison of ARID1B protein expression between normal tissue samples (n = 18) and tumor tissue samples from Luminal (n = 64), HER2+ (n = 10), and TNBC (n = 16) patients. Analysis of ARID1B protein levels using the CPTAC database and UALCAN data analysis portal showed significant accumulation of ARID1B in Luminal (p ≤ 0.0001), HER2+ (p = 0.0018), and TNBC (p ≤ 0.0001) compared to normal tissues.^[79,80]^ P-values are summarized with asterisks: *** - p ≤ 0.0001, ** - p ≤ 0.001, * - p ≤ 0.05. (C) Heatmap and hierarchical clustering analysis display row-scaled log_2_ protein expression values of 27 SWI/SNF complex-associated proteins from 56 human TNBC samples. The log2 relative protein expression scale is depicted on the top left. (D) 4X IHC of ARID1B, ARID1A, and Ki67 in breast cancer. (E) Correlation between ARID1B and ARID1A in breast cancer. (F) Kaplan-Meier survival plot for breast cancer patients with ARID1B high- (n = 606) and low- (n = 608) expressing tumors. Patients were sorted by ARID1B gene expression, and the low expression (< 10.83) was compared to the high expression (≥ 10.83). High mRNA levels of ARID1B are associated with worse prognosis in breast cancer patients. The TCGA breast cancer database and Xena platform were used for analysis.^[88]^ Time to follow-up was measured over 8,000 days. Logrank p-value = 0.0003. (G) Kaplan-Meier survival plot for breast cancer patients with ARID1A high- (n = 609) and low- (n = 605) expressing tumors. Patients were sorted by ARID1A gene expression, and the low expression (< 11.55) was compared to the high expression (≥ 11.55). There was no significant difference in survival rate between patients with high and low mRNA levels of ARID1A. The TCGA database and Xena platform were used for analysis.^[88]^ Time to follow-up was measured over 8,000 days. Logrank p-value = 0.1768. (H) Promoter methylation levels of ARID1B. The BRCA (TCGA) database and UALCAN data analysis portal were used for comparison of ARID1B promoter methylation between normal tissue (n = 97) and primary tumor (n = 793).^[79,80]^ The Beta value indicates levels of DNA methylation ranging from 0 (unmethylated) to 1 (fully methylated). The analysis showed that ARID1B promoter methylation levels were significantly lower in tumor tissue samples compared to normal tissue (p = 0.0123). P-values are summarized with asterisks: * - p ≤ 0.05. (I) Promoter methylation levels of ARID1A. The BRCA (TCGA) database and UALCAN data analysis portal were used for comparison of ARID1A promoter methylation between normal tissue (n = 97) and primary tumor tissue (n = 793).^[79,80]^ The Beta value indicates levels of DNA methylation ranging from 0 (unmethylated) to 1 (fully methylated). The analysis showed that ARID1A promoter methylation levels were significantly higher in tumor tissue samples compared to normal tissue (p = 0.0107). P-values are summarized with asterisks: * - p ≤ 0.05. (J) Comparison of cancer progenitor cell-related pathways ssGSEA enrichment score between the ARID1B low group and the ARID1B high group. All P-values are from Wilcoxon tests. *: *p* < 0.05; **: *p* < 0.01; ***: *p* < 0.001; ****: *p* < 0.0001.

**Figure S2. ARID1B-dependent transcriptional changes preferentially associate with ARID1A-mutant breast cancers. (A)** Number of differentially expressed genes (DEGs) identified between ARID1B-high and ARID1B-low breast cancer samples, including 397 significantly upregulated and 596 significantly downregulated genes. **(B), (C)** Heatmaps depicting hierarchical clustering of ARID1B-dependent **(B)** upregulated and **(C)** downregulated genes across breast cancer samples, annotated by ARID1A mutational status (mutant, *orange*; WT, *blue*). **(D), (E)** Venn diagrams illustrating the overlap between ARID1B-dependent DEGs and ARID1A-mutation-dependent DEGs: **(D)** 49 genes (10.6%) are concurrently upregulated, and **(E)** 168 genes (20.1%) are concurrently downregulated. **(F)**, **(G)** Heatmap showing hierarchical clustering specifically of the overlapping genes identified in panels D–E: **(F)**concurrently upregulated genes (49 genes) and **(G)** concurrently downregulated genes (168 genes), further demonstrating distinct transcriptional enrichment associated with ARID1A-mutant breast cancers.

**Figure S3. ARID1B KO reduces cell growth and colony formation in breast cancer cells with ARID1A mutation or loss.** (**A**) Growth rate of T47D Control and ARID1B KO (B-KO) cells**.** Cell viability was determined using the CCK-8 assay. The data is presented as the mean±SD of three independent experiments. Statistical significance was determined by two-way ANOVA. P-values are summarized with asterisks: **** - p ≤ 0.0001. **(B)** Colony formation assay in response to ARID1B KO (B-KO) in T47D cells. (**C**) Growth rate of MDA-MB-468 Control, ARID1B KO (B-KO), ARID1A KO (A-KO), and ARID1A/ARID1B KO (A/B-KO) cells. Cell viability was determined using the CCK-8 assay. The data is presented as the mean±SD of three independent experiments. Statistical significance was determined by two-way ANOVA. P-values are summarized with asterisks: ** - p ≤ 0.01, *** - p ≤ 0.001, **** - p ≤ 0.0001. **(D)** Colony formation assay in response to ARID1B KO (B-KO), ARID1A KO (A-KO), and ARID1A/ARID1B KO (A/B-KO) in MDA-MB-468 cells.

**Figure S4. ARID1B promotes colony formation in TNBC cells but does not alter sensitivity to SN38, BYL719, and GDC0914.** (**A**) Immunoblot analysis of ARID1B in wild-type (Parental), empty vector control (CRISPRv2), and ARID1B KO MDA-MB-231 cells. ARID1B KO 1-3 cell lines were each generated using different sgRNAs. GAPDH was used as a loading control. (**B**) Representative images of colony formation assay in response to overexpression and knockout of ARID1B in breast cancer cells. (**C**) Quantification of colony numbers formed in ARID1B KO MDA-MB-231 and MDA-MB-468 ARID1B OE cells compared to their corresponding empty vector controls. (**D, E, F**) Dose-response curves for three different treatments: topoisomerase I inhibitor SN38 (**D**), BYL719 (**E**), or GDC0914 (**F**) for 24, 48, and 72 hours in MDA-MB-231 empty vector control (Control) and ARID1B OE cells.

Figure S5. ARID1B modulates the therapeutic efficacy of PARP inhibitors in TNBC cells. (A), (B), (C), (D) Effect of ARID1B on the response to an IC_50_ dose of Niraparib (10µM) (A), (B) or Veliparib (64µM) (C), (D) in MDA-MB-231 (A), (C) and MDA-MB-468 (B), (D) cells. Data are presented as the mean ± SD from three independent experiments, two-tailed unpaired t-test. In the figure, “#” indicates the p-values for comparison between the specified groups. Significance levels are defined as follows: **** - p ≤ 0.0001, *** - p ≤ 0.001, ** - p ≤ 0.01, * - p ≤ 0.05.

Figure S6. ARID1B modulates efficacy of HER2-targeted drugs in HER2-positive breast cancer cells. (A), (B) Dose-response curves for HER2-targeted treatments Tucatinib (A) or Neratinib (B) for 72 hours in HCC1954 empty vector control (Control), ARID1B OE, and ARID1B KO cells. Statistical significance was determined by two-way ANOVA. P-values are summarized with asterisks: **** - p ≤ 0.0001, ** - p ≤ 0.01, * - p ≤ 0.05. (C) Immunoblot analysis of ARID1B in empty vector control (CRISPRv2) and ARID1B KO HCC1954 cells. ARID1B KO 1-3 cell lines were each generated using different sgRNAs. GAPDH was used as a loading control. (D), (E) Dose-response curves for HER2-targeted treatments Tucatinib (D) or Neratinib (E) for 72 hours in SK-BR-3 empty vector control (Control) and ARID1B OE cells. Statistical significance was determined by two-way ANOVA. P-values are summarized with asterisks: *** - p ≤ 0.001, * - p ≤ 0.05. (F), (G) Dose-response curves for HER2-targeted treatments Tucatinib (F) or Neratinib (G) for 72 hours in SK-BR-3 empty vector control (Control) and ARID1B KO cells. Statistical significance was determined by two-way ANOVA. P-values are summarized with asterisks: **** - p ≤ 0.0001. (H), (I) Colony formation assay for HER2-targeted treatments Tucatinib (H) or Neratinib (I) in HCC1954 empty vector control (Control), ARID1B OE, and ARID1B KO cells. (J), (K) Colony formation assay for HER2-targeted treatments Tucatinib (K) or Neratinib (K) in SK-BR-3 empty vector control (Control), ARID1B OE, and ARID1B KO cells.

**Figure S7.** **ARID1B overexpression promotes cancer stem cell properties in TNBC cells.** (**A), (B)** Representative images and quantification of 3D mammosphere formation assays in ARID1B OE and empty vector control (Control) MDA-MB-231 (**A**) and MDA-MB-468 (**B**) cells. ARID1B OE significantly increased the number and size of mammospheres in both cell lines. Flow cytometry analysis of CD44⁺/CD24⁻ and ALDH⁺ cancer stem cell populations in the same cells shows that ARID1B OE leads to a marked increase in stem-like subpopulations. (**C–E**) Quantification of mammosphere numbers (**C**), CD44⁺/CD24⁻ cells (**D**), and ALDH⁺ cells (**E**) in ARID1B OE versus vector control TNBC cells. Data represent mean ± SD from biological replicates; statistical significance determined by unpaired two-tailed t-test (****p < 0.0001, **p < 0.01, *p < 0.05).

**Figure S8. Nuclear accumulation of ARID1B results in a reduction of ARID1A-bound BAF complexes. (A)** Immunoblot analysis of ARID1A and SMARCE1 levels in empty vector control (pHAGE) and ARID1B OE breast cell lines. The levels of ARID1A and SMARCE1 were analyzed in SK-BR-3, BT474, and HCC1937 cell lines. GAPDH was used as a loading control. **(B)** Immunoblot analysis of ARID1A levels in empty vector control (Control) and ARID1B KO MDA-MB-468 cell lines. GAPDH was used as a loading control. **(C)** Immunoblot analysis of ARID1B levels in empty vector control (Control) and ARID1A KO MDA-MB-231 and MDA-MB-468 cell lines. Actin was used as a loading control. **(D, E)** Protein **(D)** and mRNA **(E)** levels of ARID1B in ARID1A wild-type (MCF7, ZR-75-1) and mutant (T47D) human ER+ breast cancer cell lines. ARID1B expression was significantly upregulated in ARID1A mutant cells compared to wild-type cells. Actin was used as a loading control. These results support our findings in **Figure 3** that there is a negative feedback between ARID1A and ARID1B. ARID1A mutation results in high expression levels of ARID1B. ARID1B OE results in ARID1A downregulation, while ARID1B KO results in ARID1A upregulation in breast cancer cells. The data in **(E)** are presented as mean ± SD; n = 3 independent experiments, two-tailed t-tests. **(F, G)** Cycloheximide (CHX) chase assay was performed to assess ARID1A protein stability in MDA-MB-231 ARID1B OE **(F)** and ARID1B KO **(G)** cells. Cells were treated with CHX (100 μg/mL) for the indicated time points (0–96 hours), and ARID1A levels were analyzed by immunoblotting. Actin was used as a loading control. **(H, I)** Quantification of ARID1A protein levels from immunoblots shown in panels **(F, G),** normalized to actin and plotted relative to time 0. ARID1A degradation was significantly accelerated in ARID1B OE cells compared to control, whereas ARID1A protein stability was increased in ARID1B KO cells. Data represent mean ± SD. Statistical significance was determined by two-way ANOVA. **(J)** Structure of nucleosome-bound human ARID1A-BAF complex.^[33]^

**Figure S9. RNA-seq analysis of ARID1B OE, ARID1B KO, and ARID1A KO in MDA-MB-468 cells. (A)** Differential gene expression in ARID1B OE, ARID1B KO, and ARID1A KO MDA-MB-468 cells vs. the control. (**B**) Hallmark pathway enrichment analysis of ARID1B KO versus control cells. (**C**) Hallmark pathway enrichment analysis of ARID1A KO versus control cells. (**D**) Hallmark pathway enrichment analysis of genes uniquely regulated by ARID1A KO from the ARID1A KO versus ARID1B KO comparison.

**Figure S10. ARID1B OE significantly upregulates pro-inflammatory pathways in TNBC cells. (A)** Hallmark pathway enrichment analysis of ARID1A KO and ARID1B OE shared genes. (**B**) Hallmark pathway enrichment analysis of genes uniquely regulated by ARID1B OE from the ARID1A KO versus ARID1B OE comparison. (**C**) Heatmap showing the expression of ARID1A-dependent genes in ARID1A KO and ARID1B OE MDA-MB-468 cells. (**D**) Gene set enrichment analysis (GSEA) of TNFα signaling via NFkB, interferon alpha response, interferon gamma response, IL6/JAK/STAT3 signaling hallmark pathways in ARID1B OE, ARID1B KO, and ARID1A KO MDA-MB-468 cells.

**Figure S11. Interplay between ARID1B and ARID1A in regulating metabolic pathways and cell cycle in TNBC cells. (A)** Gene set enrichment analysis (GSEA) of mitotic spindle, hypoxia, glycolysis, and mTORC1 signaling hallmark pathways in ARID1B OE, ARID1B KO, and ARID1A KO MDA-MB-468 cells. **(B)** Gene set enrichment analysis (GSEA) of the G2/M checkpoint and E2F targets hallmark pathways in ARID1B OE, ARID1B KO, and ARID1A KO MDA-MB-468 cells.

**Figure S12. ARID1B overexpression reduces ARID1A chromatin binding. (A)** ChIP-seq heatmap and average binding profiles of ARID1A peaks in Control, ARID1A KO, ARID1B KO, and ARID1B OE MDA-MB-468 cells. (**B-E**) Genome browser views showing ARID1A ChIP-seq signal near the **NT5E** (**B**), **FGF1** (**C**), **RUNX2** (**D**), and **CDKN1A**(**E**) loci across the same conditions.

**Figure S13. Identification of nuclear transport-associated proteins KPNA2, KPNB1, and RANBP2 as critical regulators of ARID1B nuclear import. (A)** The protein-protein interaction network between ARID1B and other subunits of the BAF complex identified in the mass spectrometry analysis. The image was generated by using the STRING online tool (<http://string-db.org>). **(B)** Venn diagram represents the overlapping number of proteins identified by mass spectrometry analysis after pulldown of ARID1B complexes from the nuclear fraction of MCF10DCIS.com (DCIS) and MDA-MB-231 cells. **(C)** Venn diagram represents the overlapping number of proteins between ARID1B interactome in MDA-MB-231 cells and the Tang et al. dataset. The Tang et al. dataset of differentially expressed proteins between breast tumors and adjacent non-cancerous tissue (n = 52 tissue pairs) was used in this analysis.^[51]^ **(D)** ARID1B co-localization with KPNB1. An immunofluorescence assay was conducted to detect the localization of ARID1B (*green*) and KPNB1 (*red*) in MDA-MB-468 cells. Nuclei were stained with DAPI (*blue*). **(E)** ARID1B co-localization with KPNA2 (*top*) and KPNB1 (*bottom*). An immunofluorescence assay was conducted to detect the localization of Flag-ARID1B (*green*) and either KPNA2 or KPNB1 (*red*) in ARID1B OE MDA-MB-231 cells. Nuclei were stained with DAPI (*blue*). (**F**) Proximity ligation assays showing the interaction between Flag-ARID1B and KPNA2 in ARID1B OE MDA-MB-231 cells.

**Figure S14. Disruption of KPNA2-KPNB1-RANBP2-facilitated ARID1B nuclear translocation suppresses tumor growth.** (**A**) Kaplan-Meier survival plot for breast cancer patients with KPNA2 high- (n = 1297) and low- (n = 582) expressing tumors. High mRNA levels of KPNA2 are associated with worse prognosis in breast cancer patients. The TCGA database and Kaplan Meier plotter were used for analysis.^[89]^ Time to follow-up was measured up to 300 months. Logrank p-value = 2.6e-06. (**B**) Colony formation assay in response to continuous IPZ treatment in MDA-MB-468 cells. (**C**) Survival rate of MDA-MB-468 cells under continuous IPZ treatment**.** Cell viability was determined using the CCK-8 assay. The data is presented as the mean±SD of three independent experiments. Statistical significance was determined by two-way ANOVA followed by the Bonferroni test. P-values are summarized with asterisks: *** - p ≤ 0.0001. **(D)** Cycloheximide (CHX) chase assay was performed to assess ARID1B protein stability in MDA-MB-468 cells. Cells were treated with CHX (100 μg/mL) for the indicated time points (0–72 hours), and ARID1B levels were analyzed by immunoblotting. KPNA2 served as control for fast turnover proteins. Actin served as a loading control. (**E**) Immunofluorescence assay was conducted to detect levels of ARID1B (*green*) in MDA-MB-468 cells in response to 48-hour treatment with 30µM, 40µM, and 50µM IPZ. Cells were treated with IPZ (50 µM) for 48 hours were 3x zoomed. Nuclei were stained with DAPI (*blue*). The scale bars indicate 30 µm. (**F**) Immunoblot analysis of cytosolic and nuclear fractions of MDA-MB-468 cells in response to IPZ treatment. MDA-MB-468 cells were treated with 10 µM IPZ or DMSO for 48 hours. The protein levels of ARID1B, ARID1A, and SMARCE1 in the cytosolic and nuclear fractions were examined. Lamin A/C and Histone H3 were used as nuclear marker proteins. GAPDH was used as a cytoplasmic marker protein.

**Figure S15.** **Pathway enrichment analysis for differently expressed genes in response to IPZ treatment in MDA-MB-468 cells.** (**A**) GO analysis of upregulated genes in response to IPZ treatment. (**B**) GO analysis of downregulated genes in response to IPZ treatment.

**Figure S16.** **RANBP2 KO reduces nuclear ARID1B levels in TNBC cells. (A**) Immunofluorescence assay was conducted to detect levels of ARID1B (*green*) and RANBP2 (*red*) in empty vector control (CRISPRv2) and RANBP2 KO MDA-MB-231 and MDA-MB-468 cell lines. Nuclei were stained with DAPI (*blue*). The scale bars indicate 30 µm. (**B**) Quantification of mean nuclear-to-cytosolic ARID1B fluorescence intensity in CRISPRv2 vs. RANBP2 KO breast cancer cells. (**C**) Immunofluorescence assay was conducted to detect levels of ARID1B (*green*) and SMARCA4 (*red*) in empty vector control (CRISPRv2) and RANBP2 KO MDA-MB-231 cell lines. Nuclei were stained with DAPI (*blue*). The scale bars indicate 5 µm. (**D**) Quantification of mean nuclear-to-cytosolic ARID1B fluorescence intensity in CRISPRv2 vs. RANBP2 KO MDA-MB-231 cells. (**E**) Immunoblot analysis of cytosolic and nuclear fractions of control and RANBP2 knockout (RANBP2 KO) MDA-MB-231 cells. The protein levels of RANBP2, ARID1B, ARID1A, SMARCE1, KPNB1, and KPNA2 in the cytosolic and nuclear fractions were examined. Lamin A/C and Histone H3 were used as nuclear marker proteins. GAPDH was used as a cytoplasmic marker protein.

**Figure S17. RANBP2 KO leads to proteasomal degradation of cytosolic ARID1B. (A)** Immunofluorescence assay was conducted to detect levels of ARID1B (*green*) in empty vector MDA-MB-468 cells following 48-hour treatment with 50µM MG132 or 20µM Chloroquine (CQ). Nuclei were stained with DAPI (*blue*). The scale bars indicate 30 µm. **(B)** Immunofluorescence assay was conducted to detect levels of ARID1B (*green*) and RANBP2 (*red*) in empty vector control (CRISPRv2) and RANBP2 KO MDA-MB-468 cells following 48-hour treatment with 50µM MG132. Nuclei were stained with DAPI (*blue*). The scale bars indicate 30 µm.

**Figure S18. IZ treatment abolished tumor promotion by ARID1B OE. (A)** Colony formation assay in ARID1B OE and control MDA-MB-231 cells following 2-hour treatment with 40µM IPZ. **(B)** Relative colony count of **(A)** normalized to DMSO-treated empty vector control cells. **(C)** CCK8 growth rate analysis of ARID1B OE and control MDA-MB-231 cells following 2-hour treatment with 40µM IPZ. Data represent mean ± SD from biological replicates; statistical significance determined by unpaired two-tailed t-test: ****p < 0.0001, ***p < 0.001, **p < 0.01 *p < 0.05). (**D)** Colony formation assay in ARID1B OE, ARID1B KO, and control MDA-MB-468 cells following 2-hour treatment with 40µM IPZ. (**E**) Immunoblot analysis of KPNB1 levels in KPNB1 KO and Control MDA-MB-468 cells. There was significant downregulation of KPNB1 in KPNB1 KO cells. Actin served as a loading control. **(F)** Volcano plots showing differentially expressed genes (DEGs) in KPNB1 KO MDA-MB-468 cells compared to control. (**G**) Hallmark pathway enrichment analysis of KPNB1 KO versus Control cells. (**H**) Venn diagram represents overlapping upregulated and downregulated genes between ARID1B KO and KPNB1 KO MDA-MB-468 cells.

**Figure S19. Disruption of KPNA2-KPNB1-RANBP2-facilitated ARID1B nuclear translocation alters gene expression and suppresses tumor growth.** (**A**) GO analysis of upregulated genes in response to BRMi treatment. (**B**) GO analysis of downregulated genes in response to BRMi treatment.

**Figure S20.** **Comparison between IPZ- and BRMi-induced transcriptional changes with ARID1B-dependent transcriptomes of breast cancer patients.** **(A)** Comparison of single-sample gene set enrichment analysis (ssGSEA) enrichment scores for IPZ upregulated and downregulated pathways in **Figure S15** between patients with ARID1B low levels and ARID1B high levels. All P-values are from Wilcoxon tests. *: *p* < 0.05; **: *p* < 0.01; ***: *p* < 0.001; ****: *p* < 0.0001. **(B)** Comparison of single-sample gene set enrichment analysis (ssGSEA) enrichment scores for BRMi upregulated and downregulated pathways in **Figure S19** between patients with ARID1B low levels and ARID1B high levels. All P-values are from Wilcoxon tests. *: *p* < 0.05; **: *p* < 0.01; ***: *p* < 0.001; ****: *p* < 0.0001.

**Figure S21. Protein-protein interactions between KPNB1, KPNA2, and ARID1B.** (**A**) The structure resolved for KPNB1 (*yellow*) in complex with the IBB domain A11-S54 of KPNA2 (*green*) (PDB: 1qgk). (**B**) Experimentally determined structures for mouse KPNA2 (*green*) complexed with different NLS motifs (lines in different colors, from PDB: 8fua, 6p6e, 8qxw, 8f2q). (**C**) NLS motif of ARID1B (*red*) forms multiple hydrogen bonds with KPNA2 (*green*) in the model generated for the ARID1B-KPNA2 complex. See also **Figure 8C** and **E**.

**Figure S22.** **Structural model for the interaction between RANBP2 and KPNB1. (A)** Structural model for interfacial interactions in the RANBP2-KPNB1-KPNA2-ARID1B NLS tetrameric complex. (**B**) Closeup view of the interactions between KPNB1 residues (*brown* *ball and stick*) and the five FG motifs (*blue* spheres) of RANBP2 corresponding to the region enclosed in a rectangle in panel **A**.

**Figure S23. *In silico* evaluation of the effect of double mutations on the binding free energy of the KPNA2-ARID1B complex.** The heatmaps show the change in binding free energy ∆∆G_binding_ between ARID1B and KPNA2 upon substitution of residue pairs (**A**) R1518 and D1522 and (**B**) R1518 and H1519 on the ARID1B NLS based on the *in silico* predicted structural model. The *green* boxes correspond to mutations in which the complex is destabilized by at least 0.8 kcal/mol while ΔΔG_folding_ ≤ 0.05 kcal/mol.

**Figure S24. IHC analysis of KPNA2 and RANBP2 in ARID1B OE and ARID1B KO TNBC tumors treated with Niraparib (A)** Representative 40X IHC images of KPNA2 and RANBP2 staining in ARID1B KO and Control MDA-MB-468 tumors (left panel), and in ARID1B OE and Control MDA-MB-231 tumors (right panel) following treatment with Niraparib or Vehicle (Control).
